# Decreased Circulating Glutamine and Myo-Inositol in Cervical Cancer: Exploratory NMR Metabolomics and Internal Discrimination Using Their Additive Feature (MQ)

**DOI:** 10.3390/diagnostics16213639

**Published:** 2026-11-04

**Authors:** Praveen Kumar, Nitu Nigam, Arti Gupta, Pragati Gupta, Nisha Singh, Amro Amara, Suchi Agrawal, Preeti Agrawal, Sanjay Nigam, Dibyendu Banerjee, Dinesh Kumar

**Affiliations:** 1 Cytogenetics Lab, Center for Advanced Research, King George’s Medical University, Lucknow 226003, Uttar Pradesh, India; praveenkumar08880@gmail.com; 2 Department of Zoology, Sri Awadh Raj Singh Smarak Degree College, Ram Manohar Lohia University, Gonda 224001, Uttar Pradesh, India; guptaarti2002@gmail.com; 3 Centre of Biomedical Research (CBMR), Lucknow 226014, Uttar Pradesh, India; praggupta856@gmail.com; 4 Academy of Scientific and Innovative Research (AcSIR), Ghaziabad 201002, Uttar Pradesh, India; 5 Department of Gynaecology Oncology, King George’s Medical University, Lucknow 226003, Uttar Pradesh, India; nisha.kgmc@gmail.com; 6 Protein Research Department, Genetic Engineering and Biotechnology Research Institute (GE-BRI), City of Scientific Research and Technological Applications (SRTA-City), New Borg El-Arab City P.O. Box 21934, Alexandria, Egypt; amroamara@web.de; 7 Department of Obstetrics and Gynaecology, King George’s Medical University, Lucknow 226003, Uttar Pradesh, India; obgexpert@gmail.com; 8 Department of Pathology, King George’s Medical University, Lucknow 226003, Uttar Pradesh, India; preetiagrawal@kgmcindia.edu; 9 Department of Pathology, Rama Medical College, Kanpur 209217, Uttar Pradesh, India; sknigam@yahoo.com; 10 Department of Cancer Biology, Central Drug Research Institute, Lucknow 226031, Uttar Pradesh, India; d.banerjee.cdri@csir.res.in

**Keywords:** cervical cancer, NMR-based metabolomics, glutamine, myo-inositol, MQ, age-adjusted analysis, exploratory discrimination

## Abstract

**Background**: Cervical cancer is accompanied by metabolic reprogramming, but circulating NMR metabolite signatures require rigorous statistical assessment before clinical interpretation. **Methods**: Serum metabolite concentrations were analyzed in 33 cervical cancer (CC) cases and 31 normal controls (NC). Group differences across 35 primary metabolites were assessed using Welch’s *t*-test with Benjamini–Hochberg false-discovery-rate (FDR) control; Mann–Whitney tests were used as distribution-robust sensitivity analyses. Age-adjusted linear models with HC3 robust standard errors were fitted for each primary metabolite (primary analysis: raw concentrations; sensitivity analysis: log2-transformed concentrations), with FDR correction across the 35 models. MQ was defined as [glutamine] + [myo-inositol]. ROC discrimination was summarized using stratified-bootstrap 95% confidence intervals, and paired DeLong testing compared MQ with glutamine. **Results:** Glutamine (CC 229.7 ± 102.2 μM; NC 434.8 ± 123.8 μM) and myo-inositol (CC 18.3 ± 8.8 μM; NC 35.8 ± 11.0 μM) were lower in CC, and both remained significant after age adjustment and FDR correction. Within CC, serum glutamine was strongly inversely correlated with tumor length (Spearman ρ = −0.94, *n* = 31; exploratory, requires independent replication). Apparent ROC AUCs were 0.906 for glutamine, 0.889 for myo-inositol, and 0.919 for MQ. The paired DeLong comparison of MQ versus glutamine was not statistically significant (AUC difference 0.0132; approximate 95% CI −0.0031 to 0.0295; *p* = 0.113). Repeated cross-validation is reported as within-cohort resampling performance and not independent validation. **Conclusions:** The findings support lower circulating glutamine and myo-inositol in this cohort. MQ is an exploratory additive feature without demonstrated superiority over glutamine alone; external validation and adjustment for additional clinical confounders are required.

## Simple Summary

Cervical cancer is associated with systemic metabolic alterations that may be detectable in blood. In this case–control study, serum metabolites were quantified by 800 MHz ^1^H NMR spectroscopy in 33 women with cervical cancer and 31 normal controls. Glutamine and myo-inositol were lower in the cancer group. Their derived additive feature, MQ = glutamine + myo-inositol, also showed strong apparent separation between groups. The apparent AUC difference between MQ and glutamine alone was not statistically significant in a paired DeLong comparison. Because the study comprises a single cohort without independent external validation, the findings are exploratory and do not establish clinical diagnostic or prognostic utility. Larger prospective cohorts with verified clinical covariates and external validation are required before clinical translation.

## 1. Introduction

Cervical cancer ranks as the fourth most common malignant tumor among women, with an annual global mortality exceeding 300,000 deaths [1,2]. In India, cancer prevalence is estimated at approximately 2.5 million, with about 800,000 new cases and 550,000 deaths annually [3]. According to the World Health Organization (WHO, 2022), more than 13.1 million cancer-related deaths are projected worldwide by 2030. CC remains a major public health concern, particularly in developing countries such as South Africa, India, China, and Brazil, which collectively account for more than 25% of the global CC burden. In India, CC contributes approximately 6–29% of all cancers in women across cancer registries and accounts for nearly 17% of cancer-related deaths among women aged 30–69 years [4]. Current estimates suggest that approximately 1 in 53 Indian women will develop CC during their lifetime [5]. HPV DNA is detected in approximately 99.7% of cervical cancer specimens when sensitive assays are applied to tumor tissue, which supports HPV as a necessary cause of cervical cancer [6].

Persistent infection with high-risk HPV (HR-HPV), particularly types 16 and 18, con-tributes to more than 70% of cases [7]. HR-HPV induces dysfunction of basal epithelial cells, ultimately contributing to cervical carcinogenesis [8]. The pathogenesis of CC is mainly linked to persistent HPV infection involving cells at the squamocolumnar junction of the uterine cervix. HPV is one of the most common sexually transmitted infections worldwide and is implicated in CC as well as malignancies of the anus, vulva, pharynx, and penis [9,10]. These associations highlight the need for targeted prevention strategies, including HPV vaccination and early screening programs, particularly in high-burden regions. CC development is a multifactorial process involving activation of proto-oncogenes and inactivation of tumor-suppressor genes [11].

In this context, metabolomics has emerged as a powerful approach for characterizing the biochemical landscape of CC. Metabolites are biologically active small molecules generated through metabolic processes and play pivotal roles in cellular function and disease progression [12,13]. As downstream products of diverse biological pathways, they may reflect upstream genetic alterations and environmental influences [14,15,16]. Cancer cells exhibit profound metabolic reprogramming to sustain rapid proliferation, invasion, and survival, and these metabolic alterations are increasingly recognized as important contributors to tumor progression [17].

Serum metabolomics, an emerging area of cancer research, provides insight into metabolic changes associated with CC [18]. Previous studies have identified amino acids, lipids, carbohydrates, and organic acids as potential biomarkers for CC diagnosis, prognosis, and therapeutic response [19,20]. Reported alterations in metabolites such as glutamine, lactate, sphingolipids, myo-inositol, and phospholipids are consistent with dysregulated energy metabolism, oxidative stress, and inflammatory processes in CC [21,22].

Among these metabolites, glutamine, a highly abundant nonessential amino acid synthesized by the human body, plays a pivotal role in biosynthetic pathways within proliferating cells [23]. It serves as a nitrogen source for purine and pyrimidine synthesis and as a precursor for protein and glutathione biosynthesis [24,25]. Myo-inositol (MI) is another metabolite implicated in cancer biology. It participates in cellular signaling processes, including protein kinase C-related pathways, and has been associated with proteolytic-enzyme activity observed in malignant and aggressive primary cerebral tumors [26]. Proton magnetic resonance spectroscopy (1H MRS) studies suggest that MI levels may help distinguish histological grades of brain tumors [27]. Previous studies have also reported tumor-suppressive effects of MI and its derivatives through pathways such as ERK-MAPK and PI3K-Akt, which are important for cancer-cell proliferation [28]. However, the role of MI in cancer is context dependent; it may suppress tumor growth in some settings while supporting cell proliferation in others, depending on the metabolic state and oncogenic background [28,29]. In CC, for example, SLC5A3, which encodes an MI transporter, is upregulated, and its knockdown has been reported to inhibit cancer-cell growth [30].

Taken together, previous findings suggest that glutamine and myo-inositol (MI) are involved in cancer-related metabolic processes, but their circulating levels in cervical cancer remain insufficiently characterized. The present study therefore examines serum metabolic differences between CC and normal controls using Nuclear Magnetic Resonance (NMR) spectroscopy, with particular emphasis on glutamine, MI, and their derived additive feature MQ. NMR spectroscopy enables non-destructive analysis of biological samples and provides structural and quantitative information on small molecules in complex specimens [31,32]. The statistical objective of the present analysis is to quantify group differences and evaluate within-cohort discrimination while avoiding unsupported clinical diagnostic or prognostic claims in the absence of independent external validation.

## 2. Materials and Methods

### 2.1. Sample Collection and Analysis Populations

The NMR metabolomics dataset comprised 64 observations (33 histopathologically confirmed cervical cancer cases and 31 non-cancer controls). The original clinical workbook contained 71 records (34 CC and 37 control records). Reconciliation of duplicate registrations and sample identifiers established a verified link between the 64 NMR sample IDs and the corresponding clinical records used for age adjustment. The linked NMR cohort was used for the metabolite analyses and age-adjusted models. For the subgroup analyses, cases with tumor length ≤ 1 cm are operationally designated the moderate cervical cancer group (MCC), and cases with tumor length > 1 cm are designated the severe cervical cancer group (SCC). These study-specific labels are based solely on tumor length and must not be interpreted as FIGO stage, histopathological grade, or validated clinical severity categories. All subgroup findings are exploratory.

Control participants are described as non-cancer controls as classified in the source records; available clinical laboratory results do not permit us to characterize every control as a clinically healthy volunteer. For subgroup analyses, 20 cases with tumor length ≤ 1 cm were operationally designated MCC and 13 cases with tumor length > 1 cm were designated SCC. These study-specific labels are based solely on tumor length and are not validated clinical stages, histopathological grades, or established severity categories. Age was available for all 64 linked participants. HPV status was available as a recorded entry in the source records (HPV positive in 16 of 33 CC patients and HPV negative in all 31 controls); the assay, specimen type, genotype, and timing of HPV testing were not documented in the clinical workbook. The source records do not document HPV negativity as a predefined control selection criterion. HPV status and selected other clinical variables were available in the source records, but menopausal status, major metabolic diseases, medication use, treatment status at blood collection, BMI, albumin, exact fasting status, and other potential confounders were incomplete or unavailable for comprehensive adjustment. Written informed consent was obtained from all participants. Sample collection occurred after Institutional Ethics Committee approval dated 20 June 2025. Blood was collected around breakfast time; exact fasting status and treatment status at blood draw were not available for metabolomic adjustment. Serum was obtained by centrifugation at 2000× *g* for 10 min at 4 °C and stored at −20 °C until NMR analysis. Exact storage duration and the interval between blood collection and centrifugation were unavailable.

### 2.2. Preparation of Serum Samples for ^1^H NMR Spectroscopic Analysis

Frozen serum samples were thawed before NMR preparation. For each sample, 300 µL of serum was mixed with 300 µL of deuterium oxide (D_2_O) [33,34]. The 600 µL mixture was transferred to a 5 mm NMR tube (Wilmad-LabGlass, Vineland, NJ, USA). D_2_O, TSP, and other reagents used for NMR preparation were sourced from Sigma-Aldrich (St. Louis, MO, USA). The serum-D_2_O mixture was centrifuged at 12,000× *g* for 10 min, and 550 µL of clear supernatant was transferred to a 5 mm NMR tube for analysis.

### 2.3. Acquisition of ^1^H NMR Spectra from Serum

Proton NMR spectra were acquired on an 800 MHz Bruker Avance III NMR spectrometer (Bruker BioSpin GmbH, Rheinstetten, Germany) using the Carr–Purcell–Meiboom–Gill (CPMG) spin-echo pulse sequence. Acquisition parameters followed procedures described previously [33,34,35]. Processed spectra were internally referenced to endogenous formate at δ 8.43 ppm to reduce sample-to-sample analytical variation; the formate concentration was set to 30 μM as described previously [36]. Spectra were imported into the PROFILER module of CHENOMX (version 9.0; Chenomx Inc., Edmonton, AB, Canada) for metabolite quantification. Dedicated laboratory QC samples, technical replicates, formal instrument drift/batch assessments, calibration-precision records, analyst-blinding documentation, and predefined spectral exclusion criteria were not available in the study records and cannot be reported retrospectively.

### 2.4. Statistical Analysis

**Figure 1:**
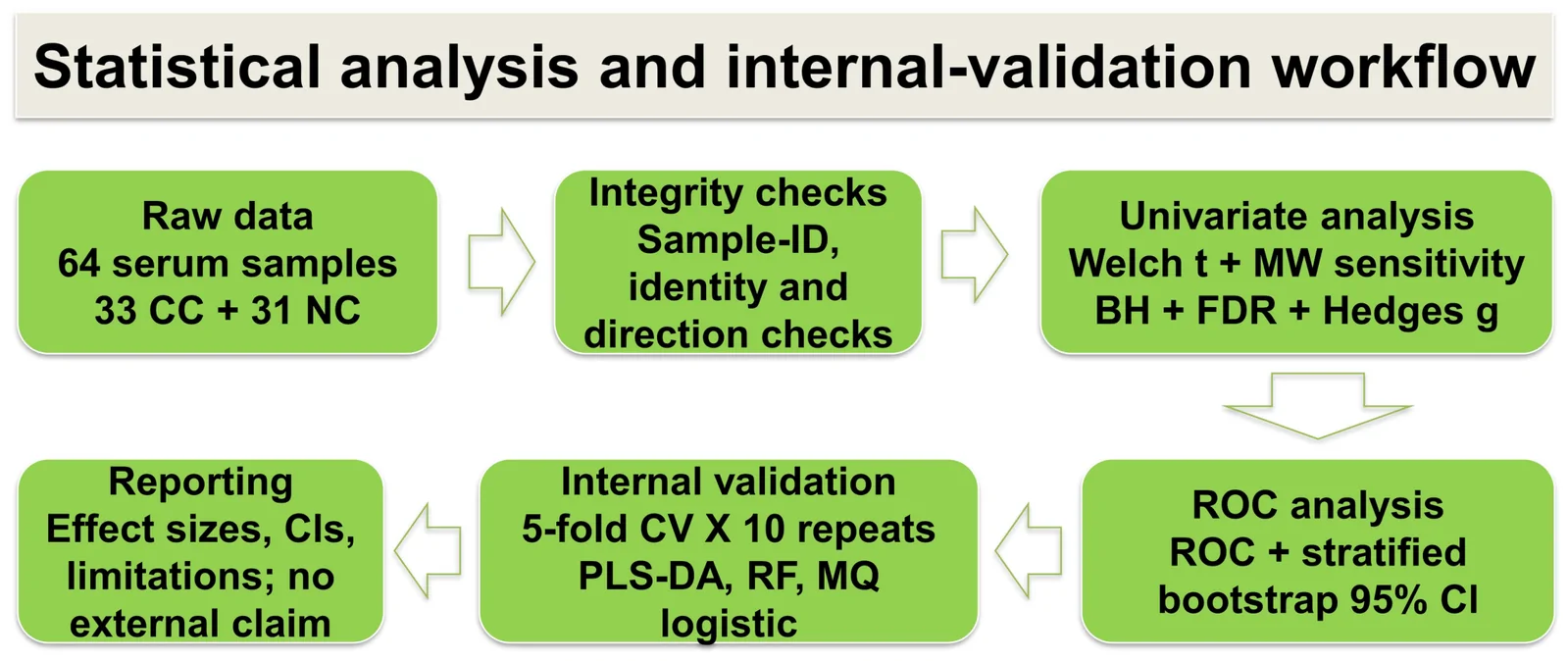
Statistical analysis and internal-validation workflow. Preprocessing and model fitting were performed within training folds to reduce information leakage.

Data integrity and analysis population. The metabolomics dataset contained 64 unique sample identifiers (33 CC and 31 NC). One aspartate value was missing; no imputation was used for univariate comparisons. For model-based analyses, median imputation was fitted within each training fold so that held-out observations did not influence preprocessing. Definition of MQ. For participant i, MQi = [glutamine]i + [myo-inositol]i, with both concentrations expressed in μM; this identity was verified across all 64 observations. MQ was treated as a derived additive feature, not as a separate metabolite. Univariate inference. Each of the 35 primary metabolites was compared between CC and NC using Welch’s two-sample *t*-test, with Benjamini–Hochberg false-discovery-rate (BH-FDR) correction across the 35 tests [37]. Mann–Whitney U tests with BH-FDR adjustment were used as sensitivity analyses. Hedges’ g (CC minus NC) quantified standardized effects. Age-adjusted analysis. For each primary metabolite, the primary model was a linear regression of raw concentration (μM) on case/control status and age. The case/control coefficient represents the age-adjusted mean difference (CC − NC, in μM). HC3 heteroscedasticity-robust standard errors [38] were used to obtain normal-approximation 95% confidence intervals and *p* values, with BH-FDR correction across the 35 models. As a sensitivity analysis, the same model was fitted to log2-transformed concentrations and expressed as an age-adjusted fold change (2^β, CC/NC). Age adjustment does not eliminate confounding by other unmeasured or incompletely documented variables. ROC analysis. ROC AUCs were calculated for glutamine, myo-inositol, and MQ, with CC as the positive class and lower values indicating CC. Stratified bootstrap resampling (4000 replicates within each group) generated 95% confidence intervals. A paired DeLong test compared the correlated ROC curves for MQ and glutamine in the same participants. Youden cutoffs, sensitivities, and specificities are exploratory in-sample estimates. Internal model assessment. Repeated stratified five-fold cross-validation with 10 repeats was used for PLS-DA and Random Forest; preprocessing was fitted within training folds. Cross-validation of the one-predictor MQ logistic model is reported as within-cohort resampling and is not interpreted as independent validation. sPLS-DA plots are exploratory because tuning and nested-validation details were not documented. Clinical covariates and tumour-length association. Clinical and demographic records were linked to NMR profiles by sample ID (Section 2.1). Clinical variables were summarized using variable-specific available *n*; continuous variables were compared by Welch’s *t*-test with BH-FDR correction across the clinical variables listed in Table 1, and categorical variables by Fisher’s exact test. Tumour-size values recorded for controls were excluded from case–control inference. Associations of serum glutamine and myo-inositol with tumour length were assessed within CC patients with a recorded tumour length (*n* = 31) using Spearman rank correlation with a bootstrap 95% confidence interval (5000 resamples), partial Spearman correlation adjusted for age, and separate analyses within the MCC and SCC groups. Age-adjusted models used raw concentrations as the primary analysis and log2-transformed concentrations as a sensitivity analysis, both with HC3 robust standard errors. Treatment status, FIGO stage, BMI, albumin, menopausal status, and other potential confounders were unavailable and could not be adjusted. Correlated ROC curves for MQ, glutamine, and myo-inositol were compared using the paired DeLong test [39]. Supplementary analyses (Figures S1–S11 and Tables S1–S6) included PCA, cross-validated PLS-DA with 1000-permutation tests [40] and VIP scores, nested cross-validation with feature selection repeated within each training fold, and exploratory analyses by tumour-size subgroup, tumour size, and HPV status. The overall analysis workflow is summarized in Figure 1.

## 3. Results

### 3.1. Cohort Reconciliation and Clinical Characteristics

The NMR analytical dataset contained 64 unique observations (33 CC and 31 NC). The original clinical workbook contained 71 records (34 CC and 37 controls); reconciliation identified duplicate UHID registrations and established sample-ID linkage for the 64 NMR observations used in the metabolomics analysis. Age was available for all 64 linked participants and was included in age-adjusted metabolite models. The clinical workbook also contained additional clinical variables with variable-specific missingness; not all were sufficiently complete or available in both groups for adjusted analysis. The cancer workbook classified cases into MCC/SCC tumor-length groups based on tumor length (MCC ≤ 1 cm, *n* = 20; SCC > 1 cm, *n* = 13). These are operational, tumor-size-based labels for exploratory comparisons only, not validated clinical stages or grades.

**Figure 2:**
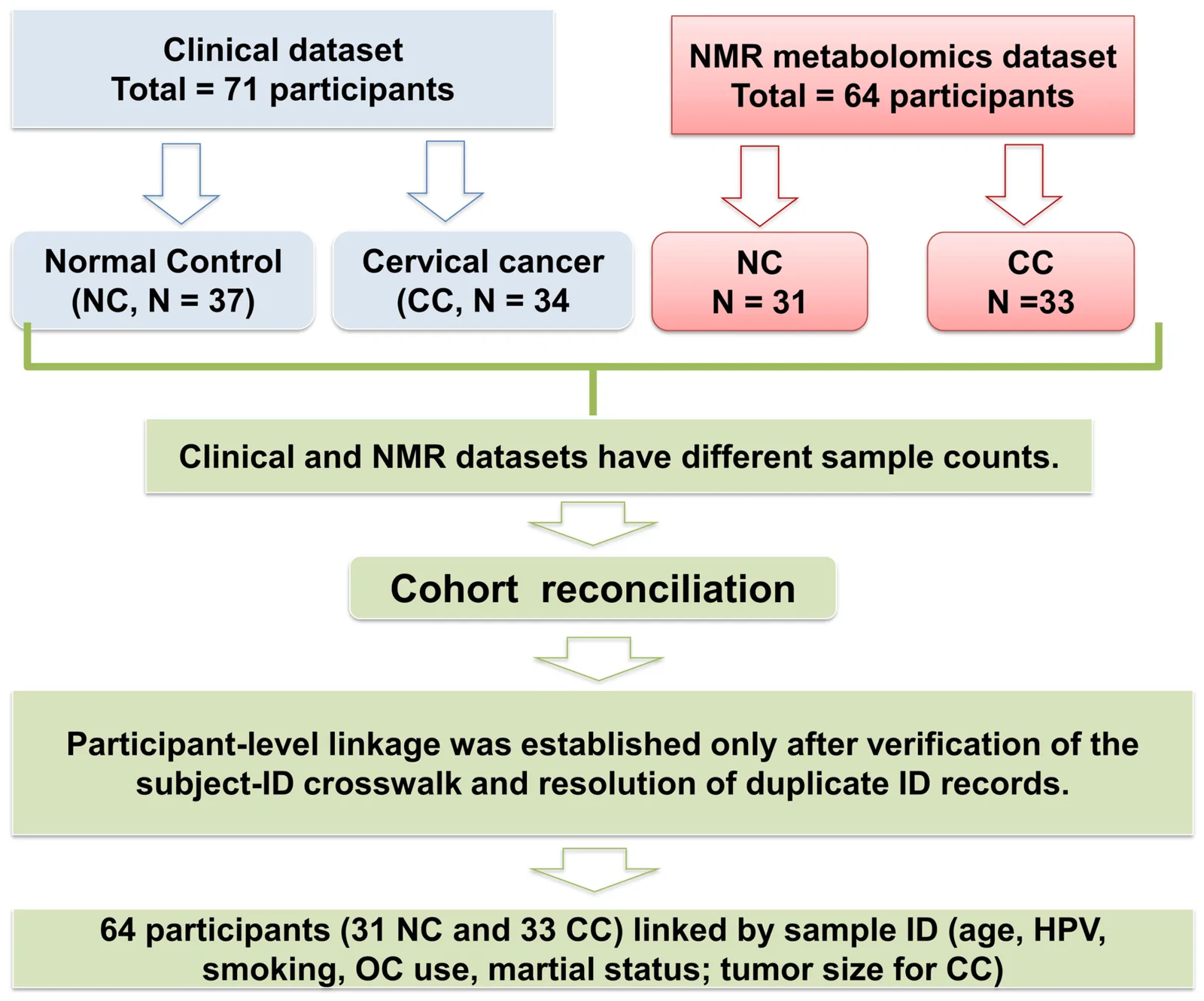
Reconciliation of the clinical and NMR analytical cohorts. The original clinical source contained 71 records (34 CC and 37 controls), while the NMR dataset contained 64 unique observations (33 CC and 31 NC). After reconciliation of duplicate registrations and sample identifiers, age was linked to the 64 NMR observations and used for age-adjusted analyses. Other clinical variables had incomplete coverage.

Clinical characteristics are summarized in Table 1 using variable-specific available *n* from the original clinical workbook. In the 64 participants linked to the NMR dataset (as per Figure 2), mean age was 52.67 ± 10.71 years in CC (*n* = 33) and 47.00 ± 11.07 years in NC (*n* = 31; Welch *p* = 0.0418). This age imbalance motivated age-adjusted linear models for each primary metabolite. Other clinical laboratory summaries in Table 1 are descriptive summaries from the source clinical workbook and have variable-specific missingness; they should not be interpreted as fully characterized covariates for all 64 NMR participants. Tumor-size fields appearing in control records were excluded from case–control inference because tumor size is not an interpretable control variable. The age-adjusted metabolite analyses are reported below and in Supplementary Table S1. HPV status was recorded as positive in 16 of 33 CC patients (48%; 10 of 20 MCC and 6 of 13 SCC) and as negative in all 31 controls (Fisher’s exact *p* < 0.001). The recorded HPV-positive proportion in CC is lower than the HPV DNA prevalence expected in histologically confirmed cervical cancer (Introduction). Because the HPV assay, specimen, genotype, and timing of testing were not documented, “negative” entries in CC patients may reflect test sensitivity, sampling or timing rather than true HPV absence, and HPV status is treated here only as a recorded clinical variable. The source records do not document HPV negativity as a predefined selection criterion for controls. Because all controls were HPV negative, HPV status could not be used as a covariate, and within CC it was not associated with glutamine, myo-inositol, or MQ (Supplementary Table S6).

### 3.2. NMR Spectra Analysis

**Figure 3:**
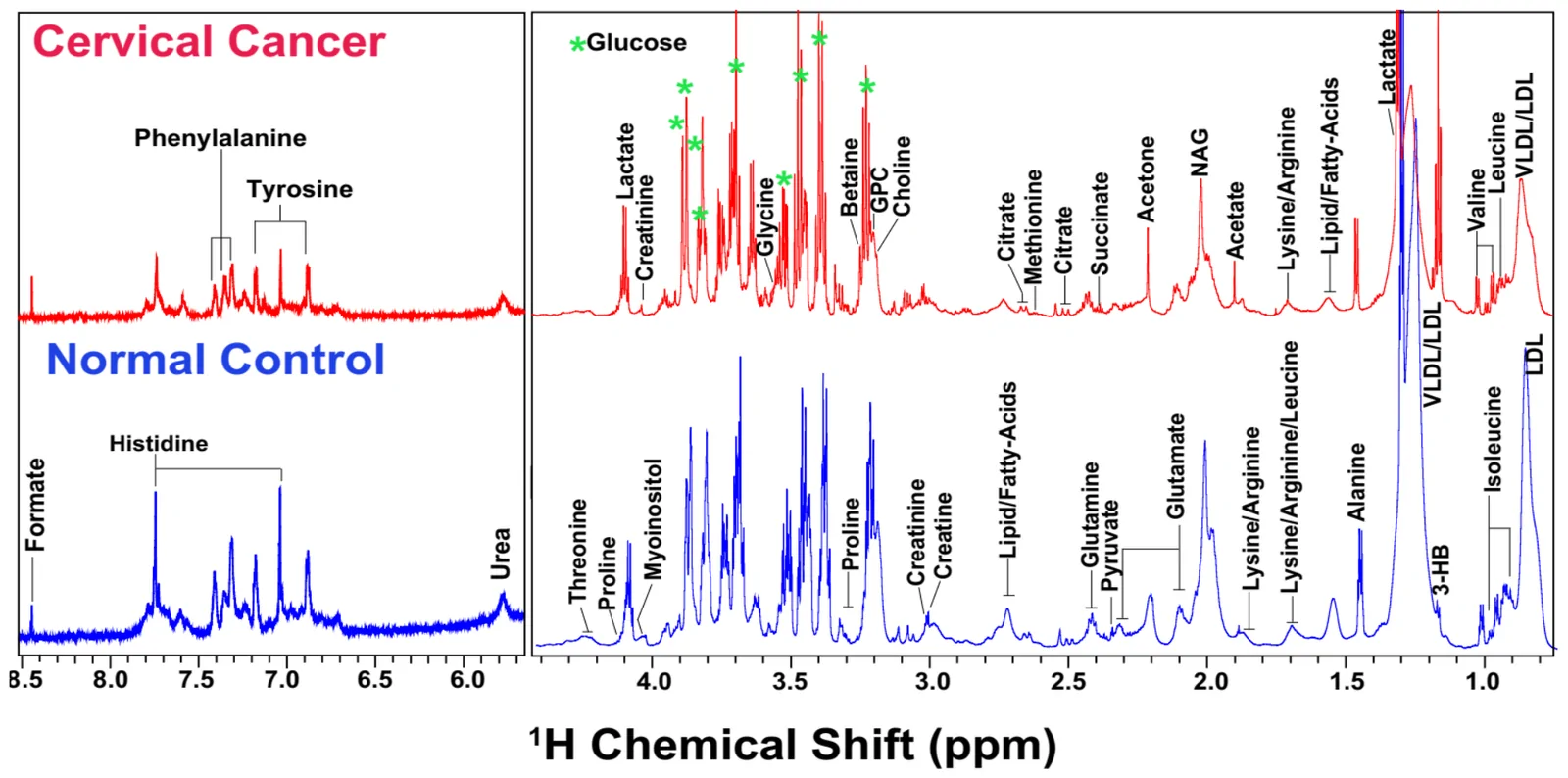
Stack plot of cumulative 1D ^1^H CPMG NMR spectra recorded from serum samples obtained from 33 CC patients (red) and 31 NC subjects (blue). Signals corresponding to glutamine and myo-inositol (MI) were identified from their characteristic chemical shifts and peak patterns using the PROFILER module of CHENOMX software (version 9.0). The *x*-axis denotes the ^1^H chemical shift (δ) in parts per million (ppm), ranging from 0.5 to 8.5 ppm. The spectra show group-associated differences across multiple metabolite regions; quantitative directionality is reported in the univariate concentration analysis.

The cumulative 1D ^1^H CPMG NMR spectra from serum samples of CC (*n* = 33) and NC (*n* = 31) showed group-associated differences in signal intensities across carbohydrates, organic acids, lipids, and amino acids (Figure 3). Peaks were annotated from chemical-shift assignments for metabolites including glucose, mannose, lactate, creatine, creatinine, MI, urea, pyruvate, succinate, alanine, valine, threonine, leucine, isoleucine, glutamate, glutamine, proline, histidine, tyrosine, phenylalanine, serine, glycine, and lysine. Because visual spectral intensity alone does not provide a reliable direction-of-change estimate for every metabolite, quantitative concentration differences were determined from the metabolite-level analyses presented below. Those analyses showed lower concentrations of glutamine, myo-inositol, tyrosine, alanine, leucine, valine, isoleucine, glutamate, glycine, and histidine in CC relative to NC. These serum spectral differences do not by themselves establish intracellular pathway flux or causality.

### 3.3. NMR-Based Clinical Metabolomics of Cervical Cancer

#### 3.3.1. Multivariate Analysis

**Figure 4:**
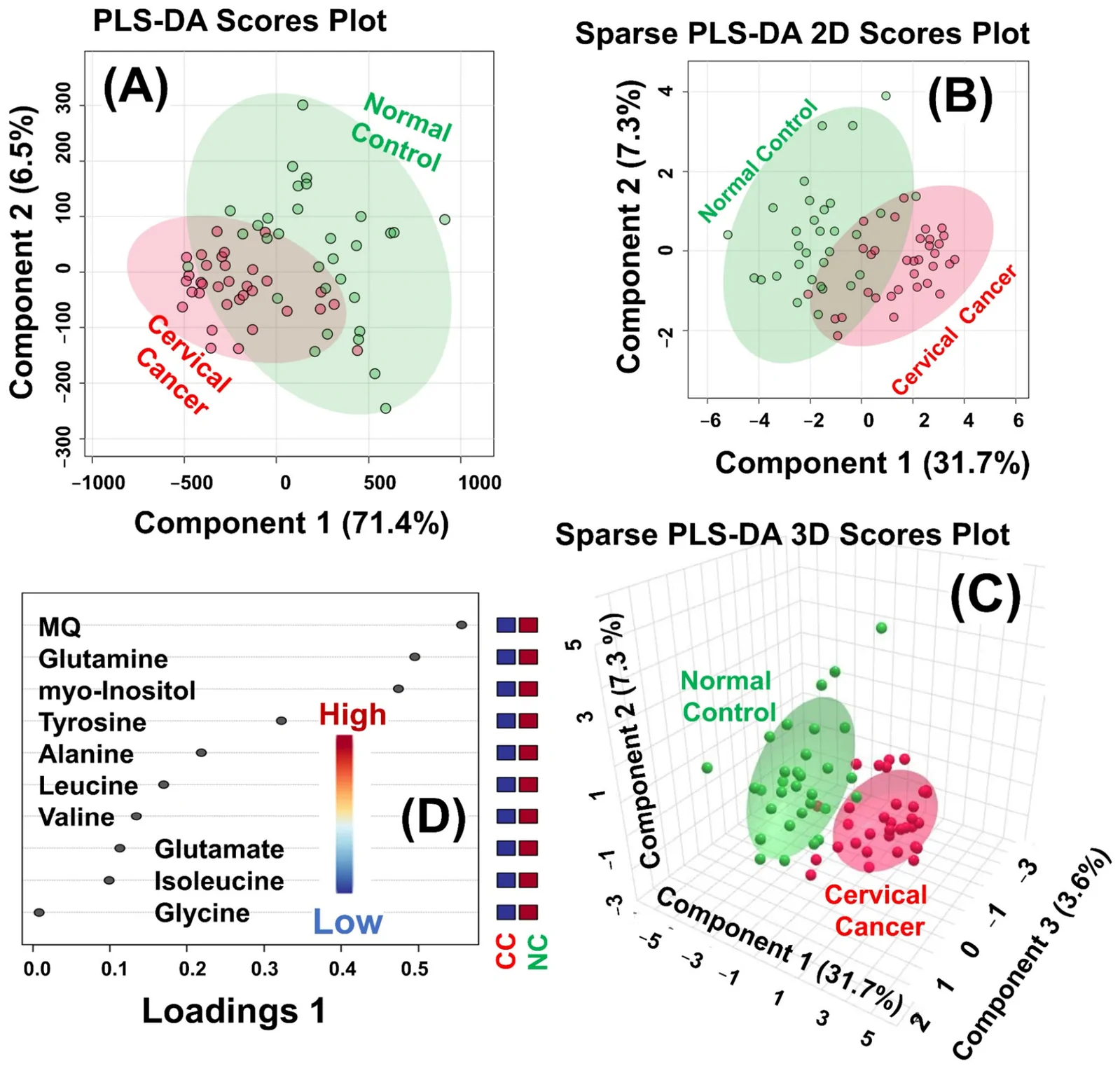
Exploratory PLS-DA and sPLS-DA analyses of serum metabolic profiles. (**A**) PLS-DA two-dimensional scores plot showing the distribution of cervical cancer (CC) and normal control (NC) samples. (**B**) sPLS-DA two-dimensional scores plot. (**C**) sPLS-DA three-dimensional scores plot. (**D**) sPLS-DA loading display showing the relative contributions of selected metabolic features. PLS-DA performance was evaluated separately using repeated held-out cross-validation; sPLS-DA visual separation is not interpreted as validated predictive performance because tuning and held-out validation details were not available.

The PLS-DA scores plot (Figure 4A) showed partial apparent separation between CC and NC samples. Sparse PLS-DA (sPLS-DA) visualizations (Figure 4B,C) showed stronger apparent separation and the loading display (Figure 4D) ranked MQ, glutamine, myo-inositol, tyrosine, alanine, leucine, and valine among the prominent contributors. However, score-plot separation in a supervised method is not evidence of validated predictive performance. PLS-DA was therefore evaluated using repeated stratified five-fold cross-validation with 10 repeats after training-fold preprocessing, yielding mean AUC 0.860 ± 0.090, balanced accuracy 0.741 ± 0.101, sensitivity 0.750 ± 0.163, and specificity 0.732 ± 0.174 [40]. The sPLS-DA plots are retained as exploratory visualizations only because the sparsity tuning grid, retained-variable settings, nested tuning procedure, and held-out performance were not documented. In supplementary analyses, the NC versus CC PLS-DA model was additionally assessed by a 1000-permutation test (R2Y = 0.61, cross-validated Q2Y = 0.34, permutation *p* = 0.001); the modest Q2Y supports its interpretation as exploratory. Principal component analysis and VIP scores are provided in Supplementary Figures S3–S5, and model summaries in Supplementary Figure S4 and Table S3.

#### 3.3.2. FDR-Controlled Univariate Analysis

**Figure 5:**
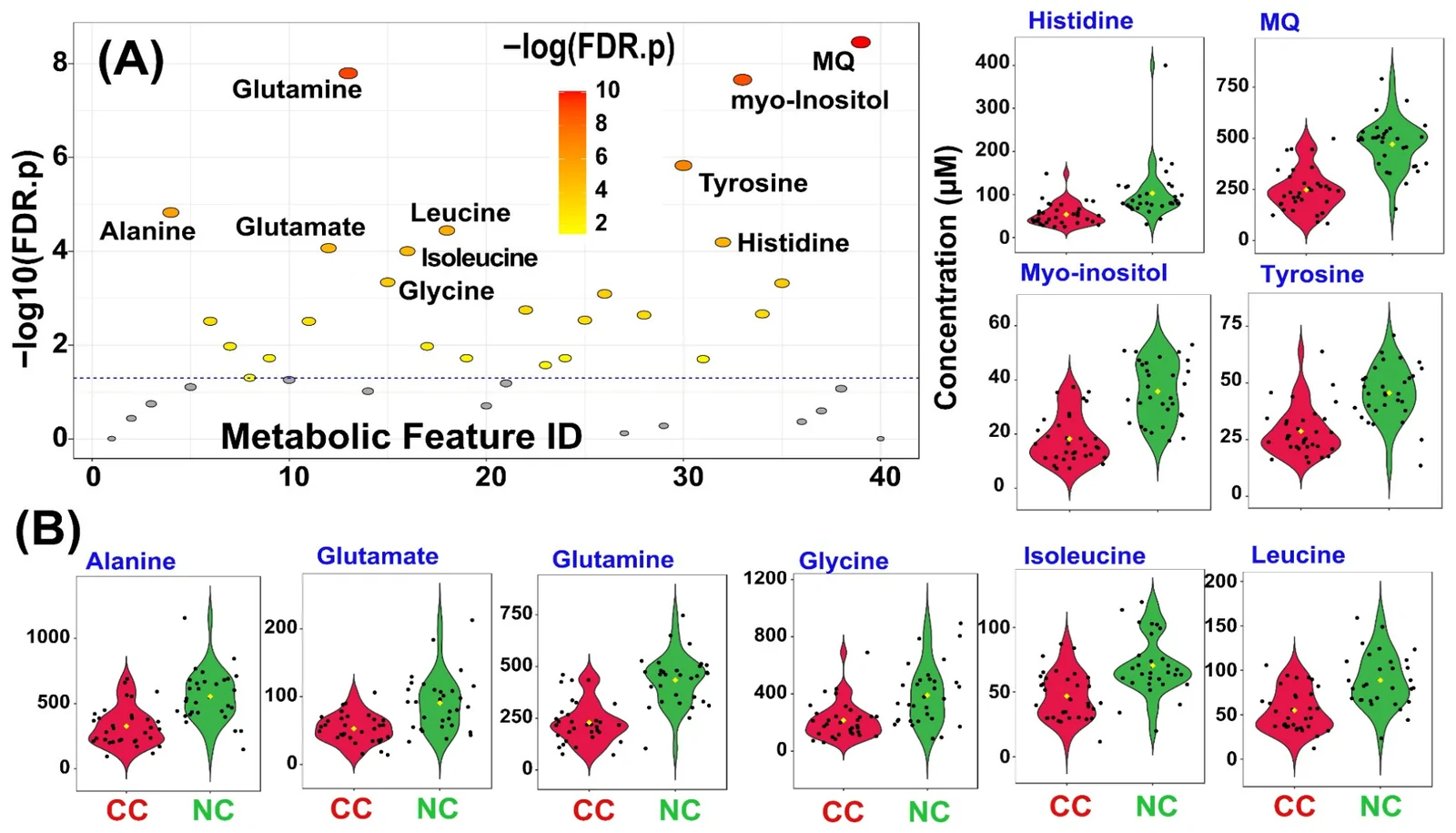
FDR-controlled univariate analysis and concentration distributions of selected serum metabolic features in cervical cancer (CC) and normal controls (NC). (**A**) Volcano plot showing the statistical significance of the univariate comparisons across the 35 primary metabolites, expressed as −log_10_ of the Benjamini–Hochberg false-discovery-rate (BH-FDR)-adjusted p-values; the dashed horizontal line indicates the significance threshold (BH-FDR-adjusted *p* = 0.05), and selected metabolites are labelled. (**B**) Violin plots showing the concentration distributions of alanine, glutamate, glutamine, glycine, histidine, isoleucine, leucine, myo-inositol, tyrosine, and the derived additive feature MQ in CC (red) and NC (green). Individual points represent participants; concentrations are expressed in μM. MQ = glutamine + myo-inositol and is a derived feature, not an independently quantified metabolite. Full univariate and age-adjusted results for all 35 primary metabolites are provided in Supplementary Table S1; additional analyses are provided in Supplementary Figures S1–S6.

Univariate comparisons showed lower concentrations of several primary metabolites in CC (see Table 2 and Figure 5). Glutamine (Hedges’ g = −1.79, approximate 95% CI −2.37 to −1.21; Welch BH-FDR q = 4.55 × 10^−8^) and myo-inositol (g = −1.75, approximate 95% CI −2.33 to −1.17; q = 4.94 × 10^−8^) showed the largest standardized differences among the highlighted features. Tyrosine, alanine, leucine, valine, isoleucine, glutamate, glycine, and histidine were also lower in CC after FDR correction. Primary age-adjusted linear models (raw concentrations, HC3 robust standard errors) showed that glutamine (adjusted difference CC − NC = −202.69 μM; 95% CI −260.59 to −144.80; q = 2.38 × 10^−10^) and myo-inositol (−17.99 μM; 95% CI −23.29 to −12.70; q = 4.90 × 10^−10^) remained significantly lower after age adjustment. In total, 22 of the 35 metabolites remained significantly different between CC and NC after age adjustment (BH-FDR q < 0.05). A sensitivity analysis on log2-transformed concentrations (HC3 standard errors) gave concordant results (age-adjusted fold change 0.50, 95% CI 0.41–0.62 for glutamine; 0.47, 95% CI 0.38–0.58 for myo-inositol; 23 of 35 metabolites with q < 0.05; Supplementary Figure S1 and Table S1). Full results for all 35 metabolites, including nominal *p*-values and FDR q-values, are provided in Supplementary Table S1.

#### 3.3.3. ROC Analysis and Internal Validation

**Figure 6:**
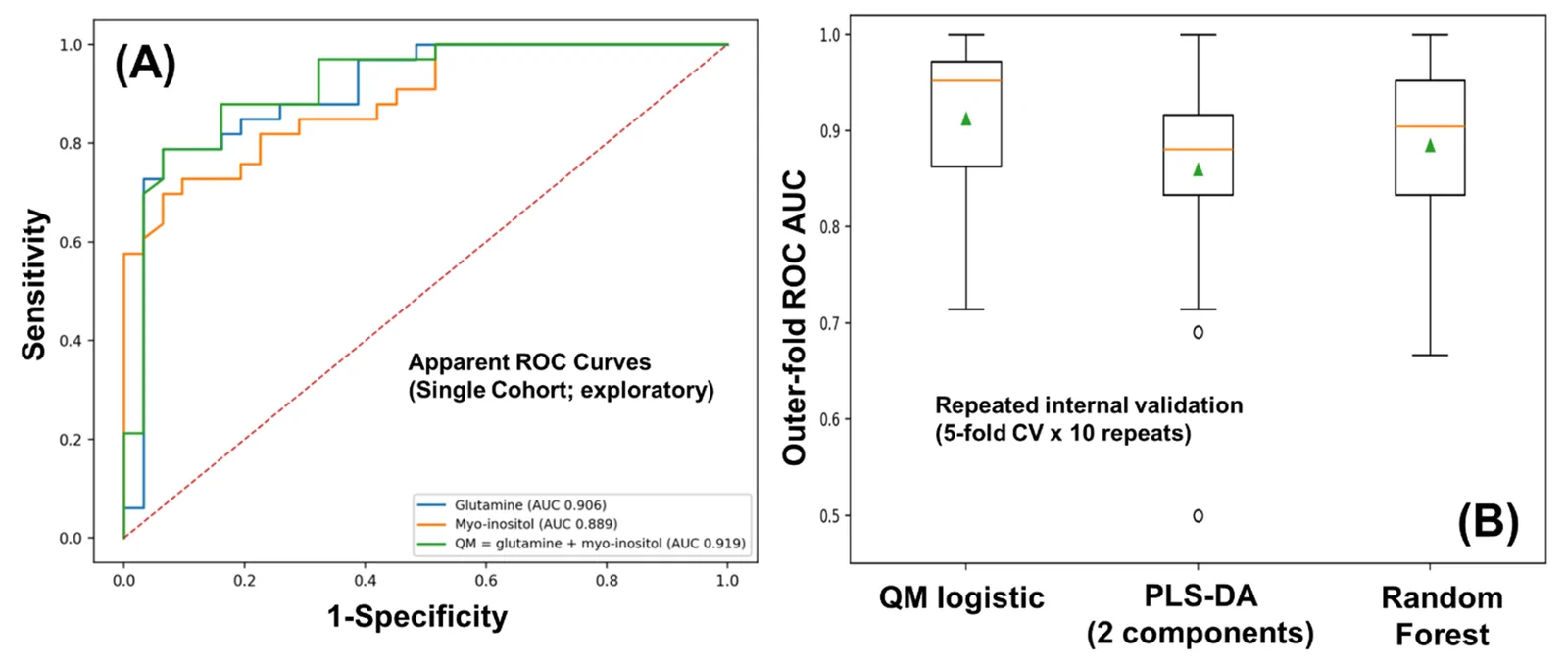
(**A**) Apparent ROC curves for glutamine, myo-inositol, and MQ. (**B**) Distribution of held-out AUC values across 50 test folds. The paired DeLong comparison of MQ versus glutamine was not statistically significant (*p* = 0.113); cross-validation estimates reflect within-cohort performance, not independent validation.

The apparent ROC AUCs were 0.906 for glutamine, 0.889 for myo-inositol, and 0.919 for MQ (Table 3 and Figure 6A). The stratified bootstrap 95% CI for MQ was 0.844–0.978. In a paired DeLong comparison using the same 64 participants, the AUC difference for MQ versus glutamine was 0.0132 (approximate 95% CI −0.0031 to 0.0295; *p* = 0.113), indicating no statistically significant difference. Because MQ is an unstandardized sum and glutamine concentrations are much higher than myo-inositol concentrations, MQ is numerically dominated by glutamine. It is therefore described as an exploratory additive feature, not a superior discriminator.

Repeated stratified five-fold cross-validation with 10 repeats yielded a mean AUC of 0.912 ± 0.078 for a one-predictor logistic model using MQ. This result reflects within-cohort resampling performance and should not be interpreted as independent validation of MQ. PLS-DA yielded mean AUC 0.860 ± 0.090, and Random Forest yielded mean AUC 0.885 ± 0.098. These estimates describe internal performance in this cohort; independent external validation is required before diagnostic utility can be assessed.

#### 3.3.4. Random Forest Classification

**Figure 7:**
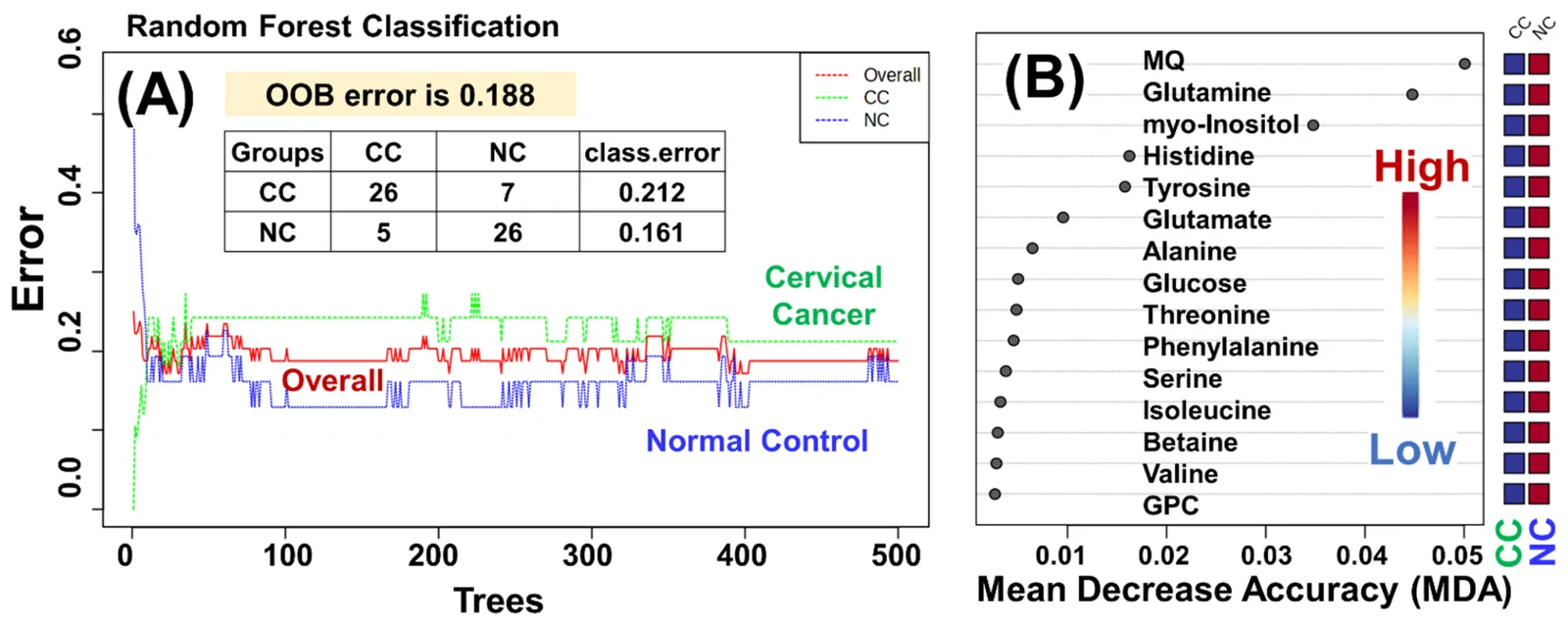
Random Forest analysis of serum metabolites distinguishing CC from NC. (**A**) OOB error profile for the fitted RF model (overall OOB error 0.188; class-specific errors 0.212 for CC and 0.161 for NC). (**B**) Mean-decrease-accuracy feature-importance ranking. OOB error and feature importance are descriptive model diagnostics; internal generalization is reported from repeated held-out cross-validation.

Random Forest (RF) was used as a nonlinear classification model and feature-ranking method. The model used 500 trees, square root feature subsampling, a minimum leaf size of 2, class-balanced weighting, and median imputation fitted within each training fold. The fitted RF model had an overall out-of-bag (OOB) error of 0.188, with class-specific errors of 0.212 for CC and 0.161 for NC (Figure 7A); these OOB values are treated as model diagnostics rather than external validation. In repeated stratified five-fold cross-validation with 10 repeats, RF achieved mean AUC 0.885 ± 0.098, balanced accuracy 0.810 ± 0.108, sensitivity 0.768 ± 0.163, and specificity 0.851 ± 0.150. The mean-decrease-accuracy plot (Figure 7B) ranked MQ, glutamine, and myo-inositol among the leading contributors, followed by histidine, tyrosine, and glutamate; these rankings describe the fitted model and do not establish causal biological importance.

#### 3.3.5. Tumor-Size Subgroups and Covariate Analyses

**Figure 8:**
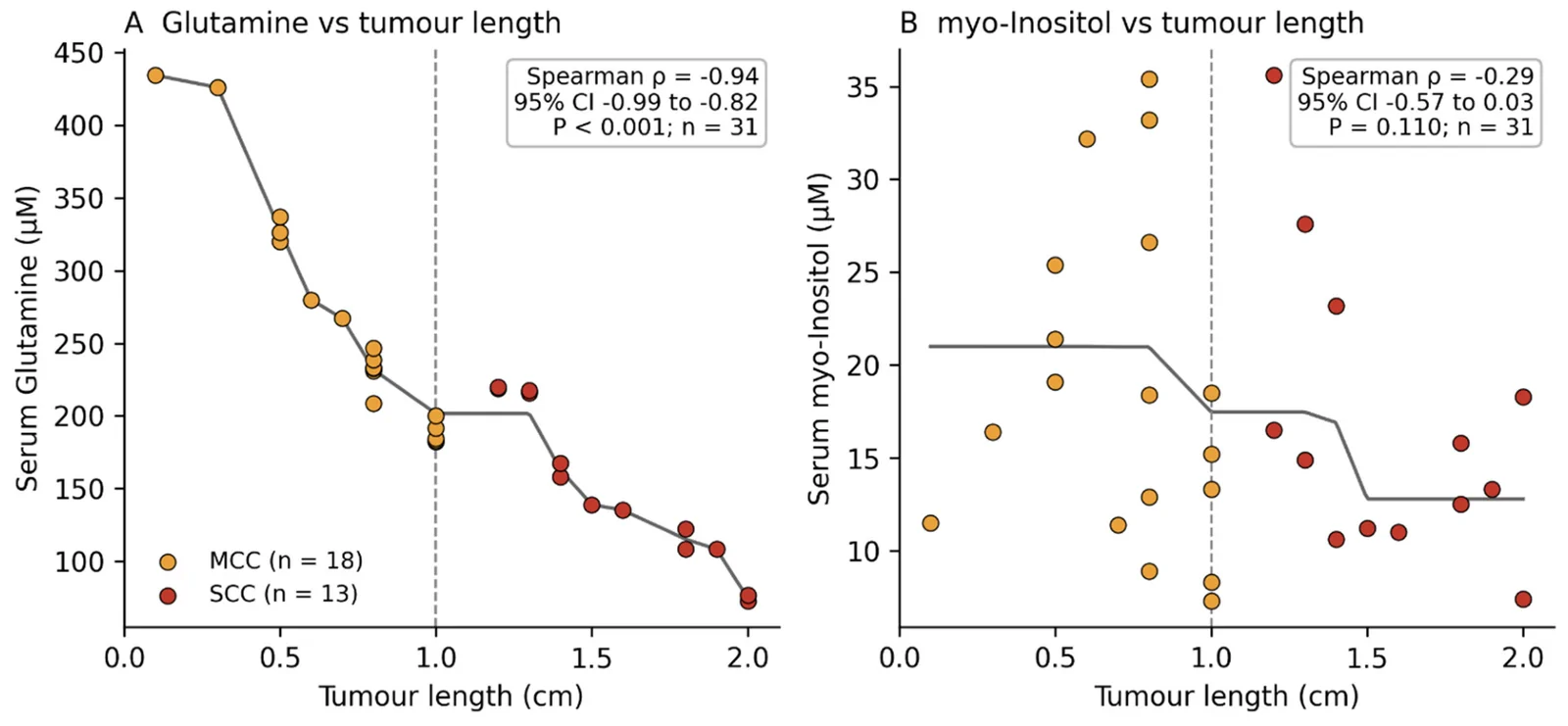
Serum glutamine and myo-inositol versus tumourlength in cervical cancer (*n* = 31 patients with a recorded tumor length: 18 MCC, 13 SCC). (**A**) Glutamine and (**B**) myo-inositol. Grey line: monotone (isotonic) fit shown for visualization only; dashed vertical line: 1-cm cut-off that defines MCC and SCC. Spearman ρ with 95% bootstrap confidence interval (5000 resamples) is shown in each panel. This is a descriptive, within-cohort association; it has not been independently validated and does not establish that glutamine predicts tumor size.

Glutamine and MQ were progressively lower across NC, MCC, and SCC (median glutamine 461, 243, and 139 µM; median MQ 498, 272, and 150 µM), and both differed between the two CC subgroups (Mann–Whitney *p* = 1.0 × 10^−4^ and 1.4 × 10^−4^, respectively; Supplementary Figure S7), whereas myo-inositol did not (median 17.4 versus 14.9 µM; *p* = 0.46). Within CC, glutamine was strongly and monotonically inversely correlated with tumor length (Spearman ρ = −0.94, 95% bootstrap CI −0.99 to −0.82, *p* < 0.001, *n* = 31; age-adjusted partial ρ = −0.94; Figure 8A), whereas myo-inositol was not significantly correlated with tumor length (ρ = −0.29, 95% CI −0.57 to 0.03, *p* = 0.110; Figure 8B). Two CC patients had no documented tumour-length measurements; their provisional MCC classification was based on metabolic features rather than measured tumour size, and they were therefore excluded from all analyses requiring confirmed tumour-length-based subgroup assignment. The glutamine–length association was also present within each tumor-size group (MCC ρ = −0.97, *n* = 18; SCC ρ = −0.99, *n* = 13; both *p* < 0.001), so it is not produced by the 1-cm grouping threshold. Glutamine was likewise inversely correlated with a tumor-volume proxy (length × width × height; ρ = −0.92), and myo-inositol weakly (ρ = −0.38, *p* = 0.034) (Supplementary Figure S10 and Table S6). The tightness of this monotone relationship is unusually high for a serum metabolite and a tumor dimension; it rests on 31 patients from a single center, the method and timing of tumor-length measurement are not recorded in the clinical workbook, and no independent replication is available. It is therefore reported as an exploratory, hypothesis-generating observation that requires verification in an independent cohort with documented measurement procedures, and it does not show that serum glutamine can predict tumor size. Because the subgroups were defined from tumor length, these observations are descriptive and do not establish an association with clinical stage or prognosis. HPV status was not associated with glutamine, myo-inositol or MQ within CC (Mann–Whitney *p* = 0.22, 0.43 and 0.14, respectively; Supplementary Table S6).

## 4. Discussion

The observed reductions in circulating glutamine and myo-inositol are consistent with a serum metabolic difference associated with cervical cancer in this cohort. However, circulating concentrations can be influenced by age, diet, fasting status, treatment, comorbidities, organ function, sample handling, and other systemic factors. In this study, age was available for all 64 NMR participants and was included in age-adjusted models; the principal reductions in glutamine and myo-inositol remained significant after adjustment. This improves the analysis but does not eliminate residual confounding because treatment status, FIGO stage, BMI, albumin, fasting status, and other relevant variables were not sufficiently documented for comprehensive adjustment.

The original clinical workbook contained 71 records, whereas the NMR dataset contained 64 unique samples. After reconciliation of duplicate UHID registrations and matching sample identifiers, age was linked to all 64 NMR observations. Mean age was higher in CC than NC in the linked cohort (52.67 ± 10.71 versus 47.00 ± 11.07 years; Welch *p* = 0.0418). We therefore fitted age-adjusted models for each of the 35 primary metabolites. The persistence of the glutamine and myo-inositol differences after age adjustment supports the robustness of these associations to age in this dataset, but does not demonstrate independence from other clinical or pre-analytical factors. The remaining clinical variables have variable-specific missingness and incomplete coverage across both groups.

HPV status was recorded as positive in 16 of 33 CC patients (48%) and as negative in all 31 controls. Because essentially all cervical cancers are HPV attributable, the recorded HPV-positive proportion in CC is lower than expected; the HPV assay, specimen, genotype, and timing of testing were not documented, so “negative” entries in CC patients may reflect test sensitivity, sampling, or timing rather than HPV-independent tumors. The source records do not document HPV negativity as a predefined selection criterion for controls. HPV status was therefore not used for modeling, and it was not associated with glutamine, myo-inositol, or MQ within CC (Supplementary Table S6). HPV-related metabolic effects cannot be assessed from these data.

PLS-DA and sPLS-DA visualizations showed apparent group structure (Figure 4), but visual separation in supervised models is not evidence of validated predictive performance. MQ is a derived feature defined as [glutamine] + [myo-inositol], not an independent metabolite. The paired DeLong test found no statistically significant difference between the apparent AUCs for MQ and glutamine (AUC difference 0.0132; approximate 95% CI −0.0031 to 0.0295; *p* = 0.113). Consequently, the present data do not demonstrate meaningful incremental discrimination from adding myo-inositol to glutamine in this unstandardized sum. MQ remains an exploratory feature requiring evaluation in larger, independent cohorts.

**Figure 9:**
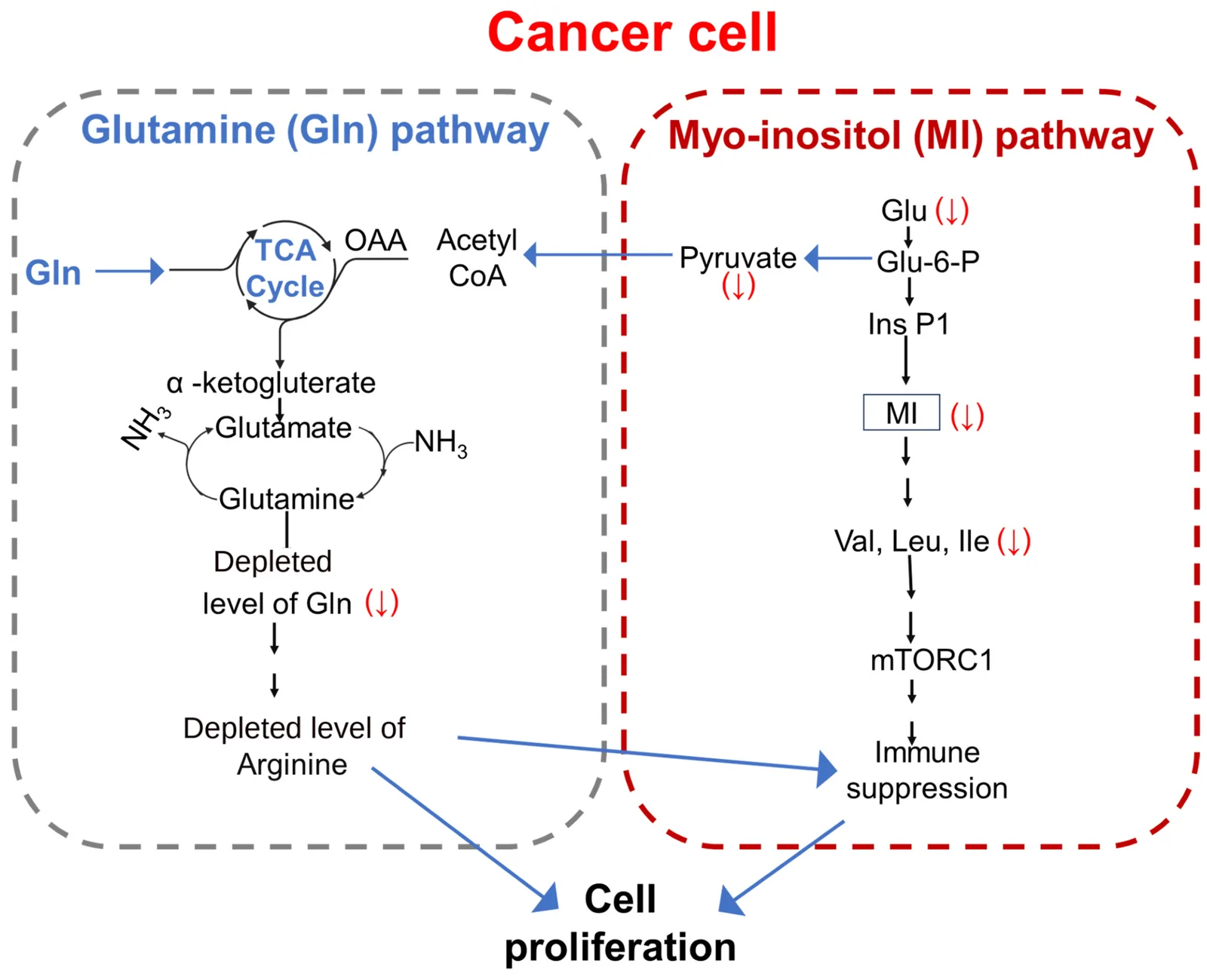
Proposed literature-informed conceptual interpretation of glutamine- and myo-inositol-related pathways in cancer cells. Downward arrows indicate the lower circulating concentrations of glutamine and myo-inositol observed in the present study. The pathway connections illustrate potential intracellular metabolic and signalling relationships discussed in the literature; they are hypothesis-generating and do not represent measured intracellular metabolic flux, causal direction, or participant-level pathway activity.

Glutamine was lower in CC than NC (229.7 ± 102.2 versus 434.8 ± 123.8 μM), and glutamate was also lower (53.2 ± 21.3 versus 90.8 ± 40.9 μM). Although glutamine metabolism is biologically important in cancer [41,42], lower serum concentrations alone cannot establish increased tumor glutaminolysis, reduced TCA-cycle input, arginine depletion, or any specific intracellular flux direction. Such mechanistic conclusions require tissue measurements, isotope tracing, flux analysis, or other direct evidence. Figure 9 is therefore a conceptual biological hypothesis rather than a measured pathway-flux model.

Myo-inositol was also lower in CC than NC (18.3 ± 8.8 versus 35.8 ± 11.0 μM). Myo-inositol participates in phosphoinositide signaling and membrane-related processes [43,44], and previous studies have investigated inositol-related mechanisms in cancer models [45,46]. In this serum dataset, the concentration difference supports an association with CC status only. It does not establish altered intracellular pathway flux, mTORC1 activity, immune suppression, or a causal mechanism. The pathway relationships shown in Figure 9 should be regarded as a literature-informed hypothesis for future mechanistic investigation.

Within CC, serum glutamine decreased monotonically with tumor length (Figure 8A), a pattern that is compatible with greater glutamine utilization by larger tumors [23,24,25] but is equally compatible with systemic factors that accompany tumor burden (nutritional status, inflammation, organ function), which could not be examined because BMI, albumin, and related variables were unavailable. The relationship was almost deterministic in this small sample and was present within both MCC and SCC; such tightness is unusual for biological data and should prompt verification of the tumor-measurement procedure and replication in independent, prospectively collected cohorts before any interpretation as a predictor of tumor size. Myo-inositol showed no significant association with tumor length (Figure 8B).

Random Forest provided complementary internal classification evidence (mean cross-validated AUC 0.885 ± 0.098), but this remains within-cohort performance rather than external validation. Feature-importance rankings are model specific and do not establish causal biological importance. Tyrosine, leucine, isoleucine, histidine, alanine, glycine, valine, and glutamate were also lower in CC after BH-FDR correction. The observed serum concentrations reflect production, consumption, transport, compartment exchange, and clearance; the present data cannot determine whether these differences result from tumor uptake, systemic metabolism, diet, treatment, fasting status, or other mechanisms. Recent work by Xu et al. reported a blood-based inflammatory biomarker incorporated into a prognostic nomogram for recurrent/metastatic cervical cancer [47]. Although that biomarker and clinical setting differ from the present study, it provides broader context for minimally invasive blood-based markers. The apparent MQ AUC was 0.919 (bootstrap 95% CI 0.844–0.978), but paired DeLong comparison with glutamine was not significant (*p* = 0.113). MQ should therefore be considered an exploratory additive feature rather than a superior discriminator. All discrimination estimates are internal to this single case–control cohort and do not establish clinical diagnostic, prognostic or predictive utility.

## 5. Limitations

This study has several limitations. First, the sample is modest (33 CC and 31 NC) and derives from a single case–control setting. Second, the clinical records were reconciled to the NMR sample IDs and age was available for all 64 participants, permitting age-adjusted analysis; however, treatment status, FIGO stage, BMI, albumin, exact fasting status, medication use, and other potential confounders were incompletely documented or unavailable for comprehensive adjustment. Third, the normal-control source records include some laboratory values outside typical reference ranges, and detailed recruitment indications and exclusion criteria could not be fully verified; therefore, the controls should be described as non-cancer controls as classified in the source records rather than assumed to be uniformly healthy volunteers. Fourth, tumor-size categories were descriptive and do not represent validated clinical stages. Fifth, ROC cutoffs and classification performance are within-cohort estimates requiring independent external validation; the paired AUC comparison did not show statistically significant superiority of MQ over glutamine. Sixth, serum concentration differences do not establish intracellular pathway flux or mechanism. Seventh, HPV status was available only as a recorded clinical-file variable (assay, specimen, genotype, and timing not documented); the recorded HPV-positive proportion in CC (16 of 33; 48%) is lower than expected for histologically confirmed cervical cancer, and all controls were recorded as HPV negative, so HPV-related effects could not be evaluated or adjusted for. Eighth, the glutamine–tumor-length association rests on 31 patients, the method and timing of tumor-length measurement were not documented, and the relationship was exceptionally tight; it requires independent replication. Finally, exact storage duration, freeze–thaw history, dedicated QC samples, technical replicates, batch distribution, instrument-drift assessment, calibration precision, analyst blinding, and predefined spectral exclusion criteria were not documented. These limitations should be addressed in larger prospective studies with standardized pre-analytical procedures, complete clinical characterization and independent validation.

## 6. Conclusions

In conclusion, glutamine, myo-inositol and several other serum metabolites were lower in the cervical cancer group than in the non-cancer control group in this cohort. The derived additive feature MQ = [glutamine] + [myo-inositol] showed an apparent AUC of 0.919 (bootstrap 95% CI 0.844–0.978). However, the paired DeLong comparison did not demonstrate statistically significant improvement over glutamine alone (AUC difference 0.0132; approximate 95% CI −0.0031 to 0.0295; *p* = 0.113). MQ is therefore an exploratory additive feature, not a validated or superior biomarker. Age-adjusted analyses support the principal glutamine and myo-inositol differences, but residual confounding and uncertainty about control recruitment remain. The strong inverse association between serum glutamine and tumor length within CC (Figure 8) is exploratory and requires independent replication. Larger prospective studies with complete clinical covariates, standardized analytical QC, and independent external validation are needed before clinical utility can be established.

## Figures and Tables

**Table 1:** Demographic and clinical characteristics of the 64 participants with linked clinical and NMR records. Variable-specific available *n* and Hedges’ g with approximate 95% confidence intervals are reported; tumor-size values from controls are excluded from case-control inference.

Variable	Control (*n*)	Control Mean ± SD	CC (*n*)	CC Mean ± SD	Welch *p*	BH-FDR q	G [95% CI]
Age (years)	31	47.00 ± 11.07	33	52.67 ± 10.71	0.0418	0.2215	0.51 [0.01, 1.01]
Tumor-size (L)	31	ND	31	1.08 ± 0.50	-	-	-
Tumor-size (W)	31	ND	31	0.77 ± 0.41	–	–	–
Tumor-size (H)	31	ND	31	0.38 ± 0.22	–	–	–
RBC (million cells/L)	6	3.79 ± 0.49	9	3.24 ± 0.59	0.0764	0.2215	−0.92 [−2.01, 0.17]
Hemoglobin (g/dL)	4	11.12 ± 1.07	9	13.63 ± 3.48	0.0771	0.2215	0.77 [−0.45, 1.99]
Hematocrit (%)	6	30.68 ± 3.65	9	26.36 ± 4.16	0.0554	0.2215	−1.02 [−2.13, 0.09]
MCV (fL)	6	71.15 ± 19.88	9	80.32 ± 6.42	0.3184	0.4308	0.65 [−0.41, 1.71]
MCH (pg)	5	27.84 ± 0.62	9	35.30 ± 17.38	0.2343	0.4014	0.49 [−0.62, 1.60]
MCHC (g/dL)	6	34.92 ± 0.92	9	34.82 ± 1.19	0.8655	0.9049	−0.08 [−1.11, 0.95]
RDW	6	15.40 ± 1.23	8	16.68 ± 2.28	0.2069	0.4014	0.62 [−0.47, 1.71]
TLC (cells/mm^3^)	6	7266.67 ± 2902.87	8	13,413.75 ± 6431.87	0.0369	0.2215	1.09 [−0.06, 2.24]
MPV (fL)	6	12.27 ± 1.18	8	10.44 ± 1.78	0.0400	0.2215	−1.10 [−2.25, 0.05]
Bilirubin (mg/dL)	5	1.51 ± 0.49	7	0.38 ± 0.15	0.0055	0.1262	−3.13 [−4.92, −1.34]
AST (IU/L)	2	74.65 ± 66.40	7	28.64 ± 12.54	0.5055	0.5813	−1.48 [−3.23, 0.27]
ALT (IU/L)	2	86.70 ± 91.36	7	19.11 ± 3.18	0.4856	0.5813	−1.73 [−3.54, 0.08]
ALP (IU/L)	2	290.35 ± 45.89	6	353.40 ± 60.97	0.2443	0.4014	0.93 [−0.76, 2.62]
Blood urea	3	26.83 ± 10.35	9	44.96 ± 27.79	0.1328	0.3394	0.66 [−0.68, 2.00]
Creatinine (mg/dL)	2	1.03 ± 0.20	9	1.52 ± 1.32	0.3183	0.4308	0.36 [−1.18, 1.90]
Sodium (mmol/L)	2	135.85 ± 0.64	10	135.74 ± 7.25	0.9634	0.9634	−0.01 [−1.53, 1.51]
Potassium (mmol/L)	2	4.55 ± 0.29	9	4.89 ± 0.51	0.3018	0.4308	0.63 [−0.93, 2.19]
Calcium (mg/dL)	2	3.36 ± 0.51	9	4.34 ± 0.73	0.1491	0.3429	1.25 [−0.39, 2.89]
Fasting glucose (mg/dL)	4	89.85 ± 12.78	6	102.37 ± 17.88	0.2336	0.4014	0.70 [−0.61, 2.01]
Postprandial glucose (mg/dL)	4	117.47 ± 17.98	6	171.29 ± 54.12	0.0615	0.2215	1.10 [−0.28, 2.48]
Prolactin (ng/mL)	6	13.07 ± 8.91	6	15.41 ± 7.32	0.6295	0.6894	0.27 [−0.87, 1.41]
Vitamin D (ng/mL)	4	30.40 ± 12.16	6	24.82 ± 2.33	0.4289	0.5480	−0.66 [−1.97, 0.65]
Marital status, married (*n*/available *n*, %)	30	28/30 (93%)	33	33/33 (100%)	0.2227	–	–
Oral-contraceptive use (*n*/available *n*, %)	31	10/31 (32%)	33	12/33 (36%)	0.7960	–	–
Smoking (*n*/available *n*, %)	31	10/31 (32%)	33	10/33 (30%)	1.0000	–	–
HPV-positive (*n*/available *n*, %)	31	0/31 (0%)	33	16/33 (48%)	3 × 10^−6^	–	–

Note: Control participants were classified as non-cancer individuals in the source records; available clinical laboratory results do not permit characterization of every control as a clinically healthy volunteer. Tumor-size values appearing in control records were non-applicable source-data entries and were excluded from case–control inference. Variable-specific available *n* and Hedges’ g with approximate 95% confidence intervals are reported. Categorical variables are shown as *n*/available *n* (%) and were compared by Fisher’s exact test (*p* value shown in the Welch *p* column). BH-FDR q values were recalculated across the clinical variables listed in the table. HPV status was recorded as negative for all controls.

**Table 2:** FDR-controlled univariate analysis of primary serum metabolites. Hedges’ g is calculated as CC minus NC; negative values indicate lower concentrations in CC. Approximate 95% confidence intervals are shown for Hedges’ g.

Metabolite	CC Mean ± SD	NC Mean ± SD	g [95% CI]	Welch *p*	BH-FDR q	MW-FDR q
Glutamine	229.7 ± 102.2	434.8 ± 123.8	−1.79 [−2.37, −1.21]	1.30 × 10^−9^	4.55 × 10^−8^	8.66 × 10^−7^
myo-Inositol	18.3 ± 8.8	35.8 ± 11.0	−1.75 [−2.33, −1.17]	2.82 × 10^−9^	4.94 × 10^−8^	1.63 × 10^−6^
Tyrosine	28.6 ± 10.7	45.6 ± 12.1	−1.46 [−2.01, −0.91]	1.79 × 10^−7^	2.09 × 10^−6^	9.43 × 10^−6^
Alanine	327.3 ± 151.8	555.4 ± 193.6	−1.30 [−1.84, −0.76]	2.58 × 10^−6^	2.25 × 10^−5^	2.85 × 10^−5^
Leucine	54.8 ± 23.8	88.7 ± 30.4	−1.23 [−1.77, −0.69]	7.21 × 10^−6^	5.05 × 10^−5^	1.75 × 10^−4^
Valine	127.5 ± 57.6	204.1 ± 70.3	−1.18 [−1.71, −0.65]	1.38 × 10^−5^	8.03 × 10^−5^	1.67 × 10^−4^
Isoleucine	46.9 ± 18.3	70.4 ± 22.7	−1.13 [−1.66, −0.60]	2.75 × 10^−5^	1.37 × 10^−4^	1.75 × 10^−4^
Glutamate	53.2 ± 21.3	90.8 ± 40.9	−1.15 [−1.68, −0.62]	3.76 × 10^−5^	1.65 × 10^−4^	2.38 × 10^−4^
Glycine	215.7 ± 129.2	392.0 ± 206.5	−1.02 [−1.54, −0.50]	1.72 × 10^−4^	6.67 × 10^−4^	3.87 × 10^−4^
Histidine	53.8 ± 25.3	103.0 ± 64.3	−1.01 [−1.53, −0.49]	2.90 × 10^−4^	1.02 × 10^−3^	8.93 × 10^−6^

**Table 3:** Apparent ROC discrimination for the prespecified reporting features. Cutoff, sensitivity, and specificity are exploratory in-sample estimates and are not externally validated operating characteristics. The circulatory levels of glutamine, myo-inositol and MQ cutoffs are expressed in μM.

Feature	AUC	Bootstrap 95% CI	Youden Cutoff	Sensitivity	Specificity
Glutamine	0.906	0.817–0.973	279.9	0.788	0.935
Myo-inositol	0.889	0.804–0.955	19.1	0.697	0.935
MQ	0.919	0.844–0.978	312.1	0.788	0.935

## Data Availability

Raw NMR spectral data will be made available on request to the corresponding author (Dinesh Kumar), subject to institutional data-sharing approval. Group-level results for all 35 primary metabolites are provided in Supplementary Table S1.

## Supplementary Materials

The following supporting information can be downloaded at: https://www.mdpi.com/article/10.3390/diagnostics16213639/s1. Figure S1: Age-adjusted differences in all 35 primary serum metabolites between CC and NC; Figure S2: Age distribution and relationship of age with glutamine and myo-inositol; Figure S3: Principal component analysis (PCA) of serum metabolic profiles; Figure S4: Cross-validated PLS-DA models and permutation tests; Figure S5: Variable importance in projection (VIP) for the NC versus CC PLS-DA model; Figure S6: Volcano plot of NC versus CC; Figure S7: Serum glutamine, myo-inositol and MQ across NC and the two tumour-size subgroups; Figure S8: ROC curves for glutamine, myo-inositol and MQ; Figure S9: Metabolite ratio features; Figure S10: Relationship between tumour size and serum glutamine or myo-inositol within CC; Figure S11: Fold-change summary across group comparisons; Table S1: Complete results for all 35 primary serum metabolites (CC versus NC); Table S2: Demographic and clinical characteristics by group (linked records); Table S3: Cross-validated PLS-DA summary; Table S4: ROC analysis, paired DeLong comparisons and cross-validated performance; Table S5: Nested internal validation; Table S6: Tumour size and HPV status in relation to glutamine, myo-inositol and MQ within CC.

## Abbreviations

CC	Cervical Cancer
NC	Normal Control
MCC	moderate cervical cancer group (tumor length ≤ 1 cm; operational tumor-size label only, not a validated stage or grade)
SCC	severe cervical cancer group (tumor length > 1 cm; operational size-based label only)
MQ	Derived additive feature defined as [glutamine] + [myo-inositol]
NMR	Nuclear Magnetic Resonance
^1^H NMR	Proton Nuclear Magnetic Resonance
^1^H MRS	Proton Magnetic Resonance Spectroscopy
CPMG	Carr-Purcell-Meiboom-Gill (pulse sequence)
TSP	Trimethylsilyl Propionic Acid (NMR internal standard)
D_2_O	Deuterium Oxide
PLS-DA	Partial Least Squares Discriminant Analysis
sPLS-DA	Sparse Partial Least Squares Discriminant Analysis
ROC	Receiver Operating Characteristic
AUC/AUROC	Area Under the ROC Curve
CI	Confidence Interval
BH-FDR	Benjamini–Hochberg False Discovery Rate
HC3	Heteroscedasticity-consistent covariance estimator (MacKinnon–White type 3)
PCA	Principal Component Analysis
UHID	Unique Hospital Identification
VIP	Variable Importance in Projection
RF	Random Forest
OOB	Out-of-Bag (error rate in random forest)
MCV	Mean Corpuscular Volume
MCH	Mean Corpuscular Hemoglobin
MCHC	Mean Corpuscular Hemoglobin Concentration
RDW	Red Cell Distribution Width
TLC	Total Leukocyte Count
MPV	Mean Platelet Volume
RBC	Red Blood Cells
Hb	Hemoglobin
Hct/PCV	Hematocrit/Packed Cell Volume
AST (SGOT)	Aspartate Aminotransferase (Serum Glutamate Oxaloacetate Transaminase)
ALT (SGPT)	Alanine Aminotransferase (Serum Glutamate Pyruvate Transaminase)
ALP	Alkaline Phosphatase
Na^+^	Sodium
K^+^	Potassium
MI	Myo-inositol
HPV	Human Papillomavirus
HR-HPV	High-Risk Human Papillomavirus
mTOR	Mammalian Target of Rapamycin
BCAAs	Branched-Chain Amino Acids
ROS	Reactive Oxygen Species
