# Supplementary material for: Decreased Circulating Glutamine and Myo-Inositol in Cervical Cancer: Exploratory NMR Metabolomics and Internal Discrimination Using Their Additive Feature (MQ)
Source: Diagnostics (Basel). 2026 Nov 4;16(21):3639. doi: 10.3390/diagnostics16213639 (PMC13762173; doi:10.3390/diagnostics16213639)
Supplement: Supplementary file 1 [file diagnostics-16-03639-s001.zip › diagnostics-4595729-supplementary/diagnostics-4595729-supplementary.pdf]

Supplementary Information

Decreased Circulatory Glutamine and Myo-Inositol in Cervical Cancer: Exploratory NMR Metabolomics and Internal Discrimination Using Their Additive Feature (MQ)

Scope and conventions

This file contains supplementary figures (Figures S1–S11) and supplementary tables (Tables S1–S6) supporting the revised manuscript. Clinical and demographic records were linked to NMR sample identifiers for all 64 participants (33 cervical cancer, CC; 31 normal controls, NC). Within CC, two tumour-size-based groups are used: moderate cervical cancer (MCC; tumour length ≤1 cm; n = 20) and severe cervical cancer (SCC; tumour length >1 cm; n = 13). These labels denote tumour-size categories used for descriptive purposes only and are not validated clinical stages or grades; tumour length was unavailable for two MCC patients. Metabolite concentrations are in μM. Unless stated otherwise, multivariate analyses used log10-transformed, autoscaled concentrations of the 35 primary metabolites, and multiplicity control used the Benjamini–Hochberg false-discovery-rate (BH-FDR) procedure. All analyses are exploratory and internal to a single cohort; they do not constitute external validation.

Supplementary Figures

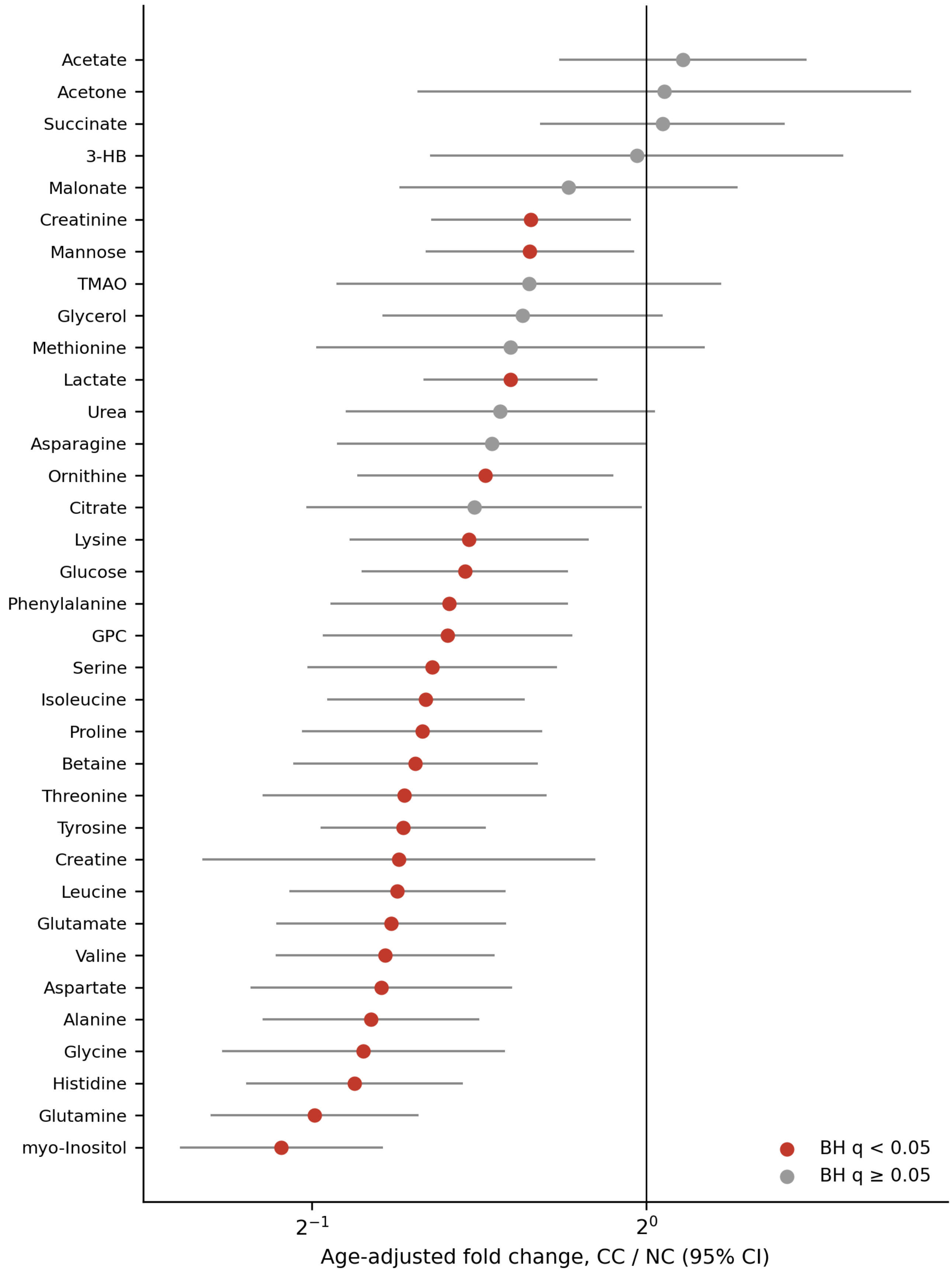

Figure S1. Age-adjusted differences in all 35 primary serum metabolites between CC and NC. For each metabolite, log2 concentration was regressed on group (CC versus NC) and age ($n = 64$: 33 CC, 31 NC). Points show the age-adjusted fold change (CC/NC) with 95% confidence intervals (log2 axis). Red points: BH-FDR $q < 0.05$ across the 35 metabolite tests (24 of 35); grey points: $q \geq 0.05$. Values are tabulated in Table S1.

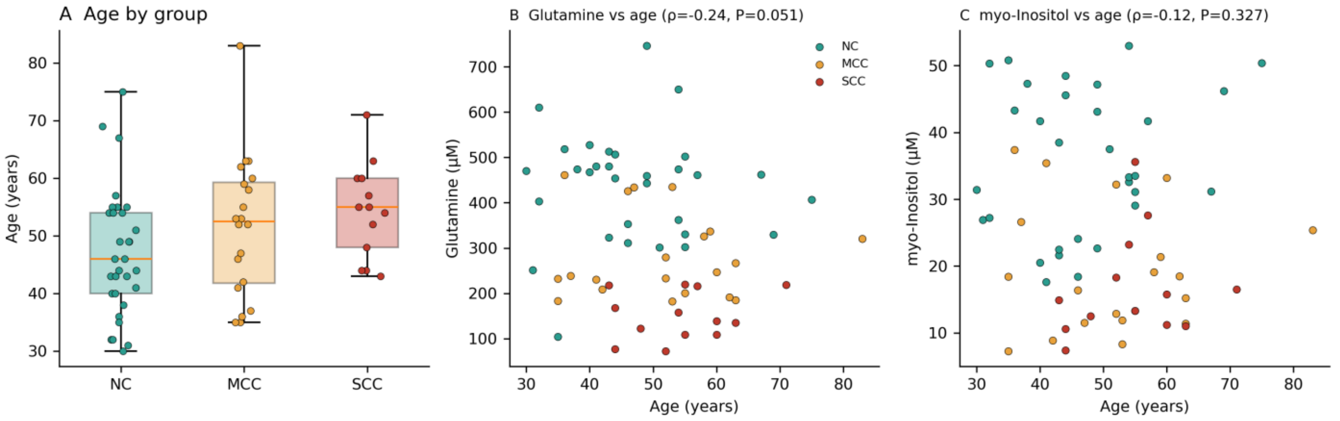

Figure S2. Age distribution and relationship of age with glutamine and myo-inositol. (A) Age by group (box: median and interquartile range; whiskers: 1.5 × IQR; points: individual participants). (B, C) Serum glutamine and myo-inositol versus age; Spearman correlation coefficients (ρ) and P values are given in the panel titles (all 64 participants). Colours: NC (teal), MCC (amber), SCC (red).

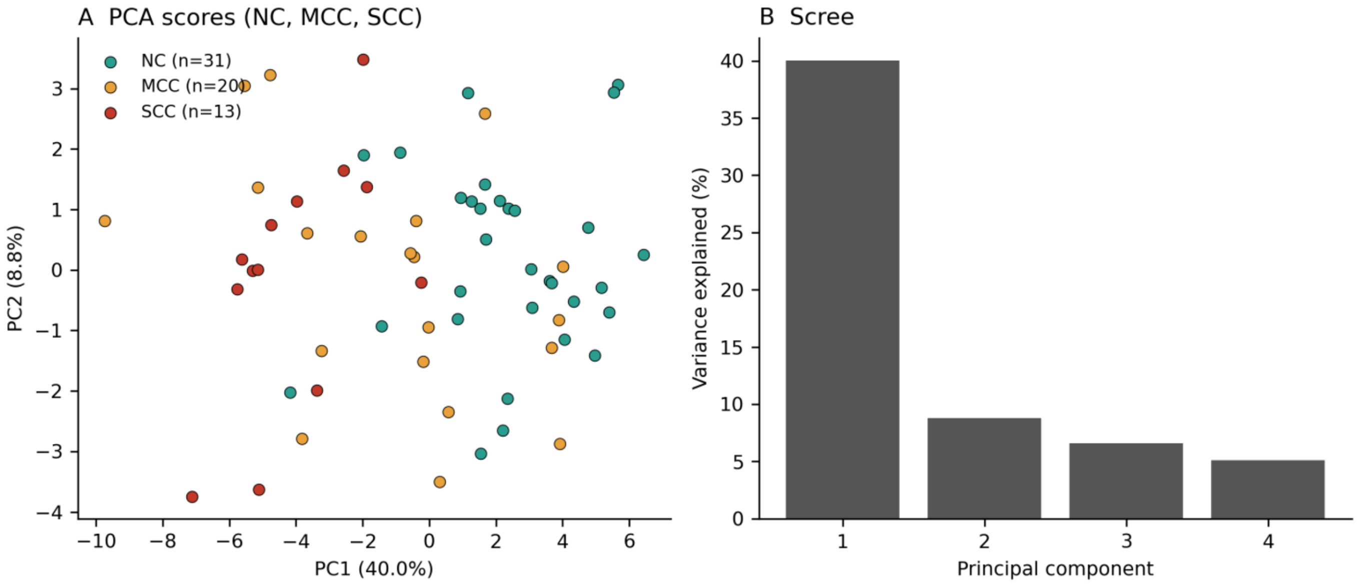

Figure S3. Principal component analysis (PCA) of serum metabolic profiles. (A) PCA scores plot (PC1 versus PC2) for NC, MCC and SCC, using the 35 log10-transformed, autoscaled metabolites. (B) Percentage of variance explained by the first four components. PCA is unsupervised and shown for exploratory visualization of overall data structure.

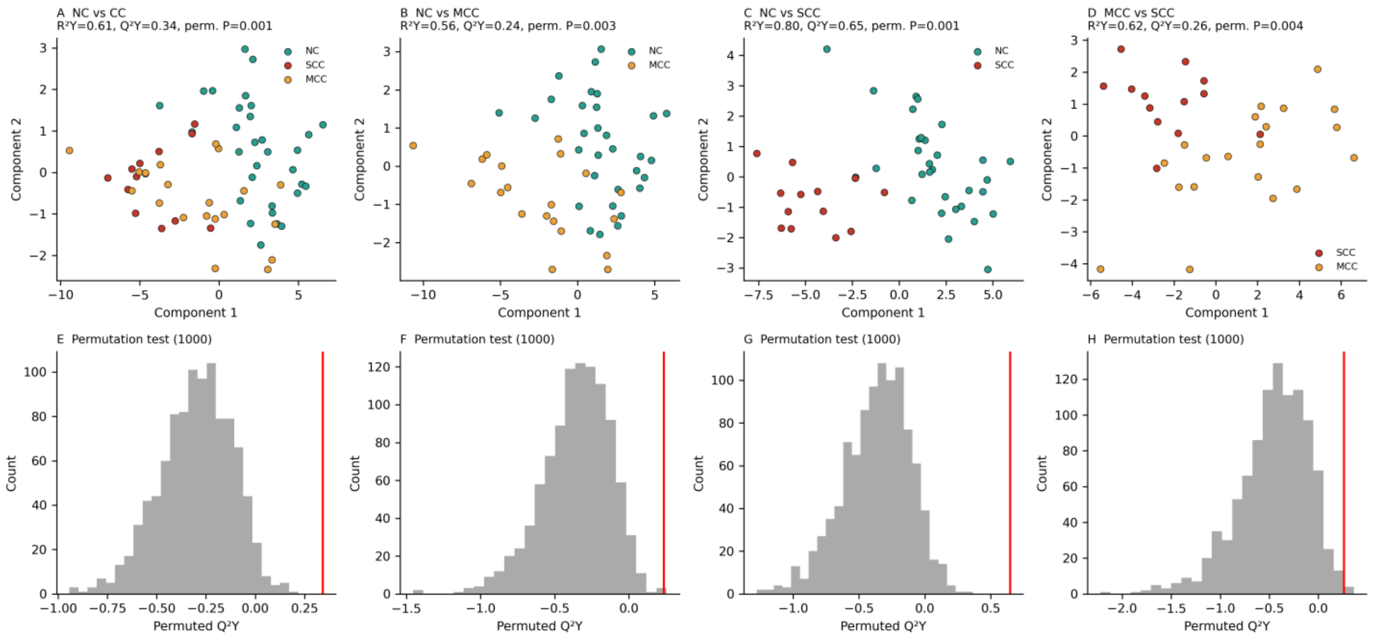

Figure S4. Cross-validated PLS-DA models and permutation tests. (A–D) Two-component PLS-DA scores plots for NC versus all CC, NC versus MCC, NC versus SCC, and MCC versus SCC. Panel titles give the fitted R^2Y, the cross-validated Q^2Y (repeated stratified five-fold cross-validation, five repeats) and the permutation P value. (E–H) Null distributions of Q^2Y from 1,000 class-label permutations (grey); the red line marks the observed Q^2Y. Q^2Y was modest for NC versus CC (0.34), NC versus MCC (0.24) and MCC versus SCC (0.26), and higher for NC versus SCC (0.65). Score-plot separation alone is not interpreted as validated predictive performance.

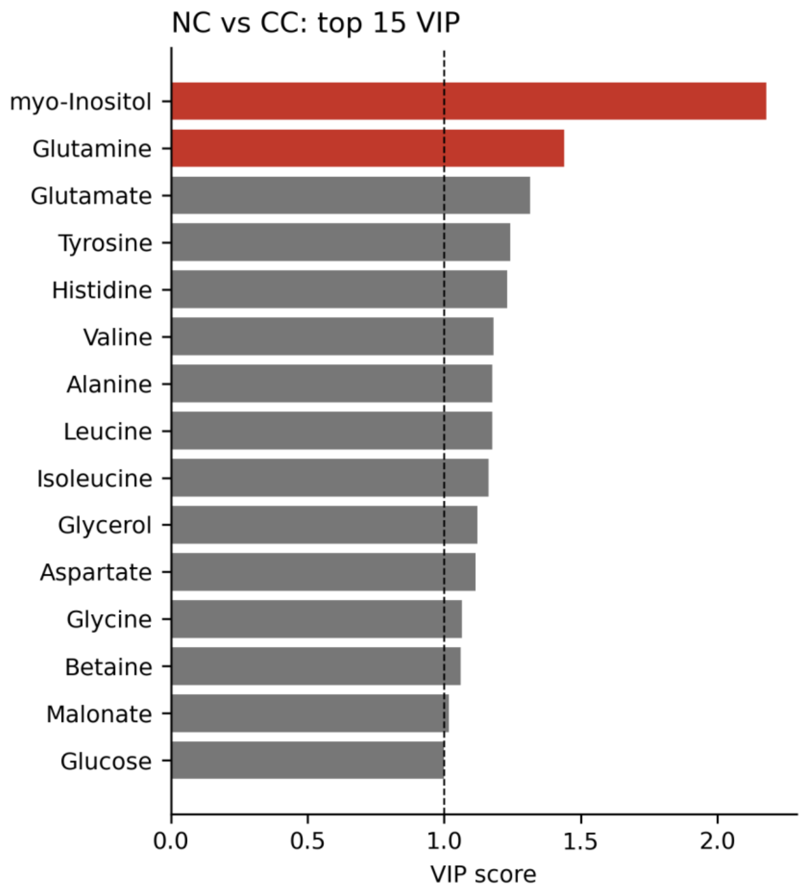

Figure S5. Variable importance in projection (VIP) for the NC versus CC PLS-DA model. The 15 metabolites with the highest VIP scores are shown; glutamine and myo-inositol are highlighted in red. The dashed line marks VIP = 1.

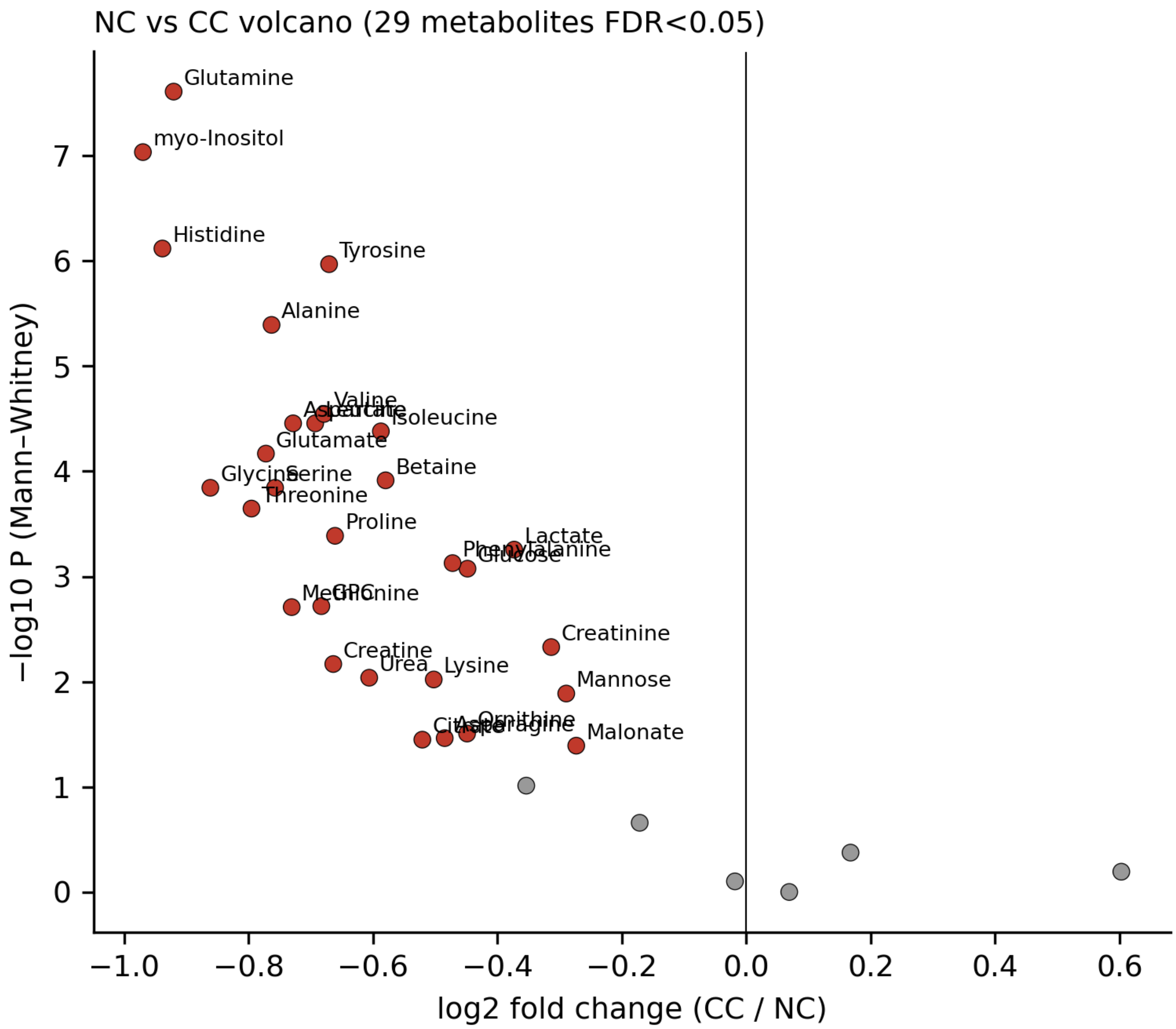

Figure S6. Volcano plot of NC versus CC. The x-axis shows the log2 ratio of mean concentrations (CC/NC) and the y-axis shows −log10 P (Mann–Whitney U test) for each of the 35 metabolites. Red points: BH-FDR q < 0.05 (labelled).

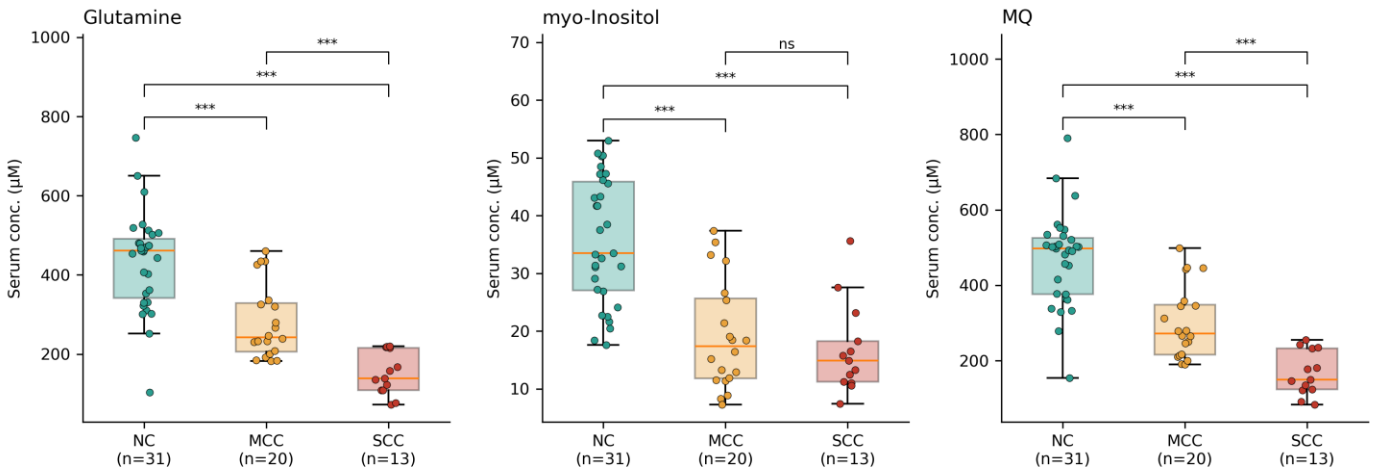

Figure S7. Serum glutamine, myo-inositol and MQ across NC and the two tumour-size subgroups. Boxes show the median and interquartile range, whiskers the 1.5 × IQR range, and points individual participants. Brackets show pairwise Mann–Whitney U tests (unadjusted): ns, P ≥ 0.05; *, P < 0.05; **, P < 0.01; ***, P < 0.001. MQ = glutamine + myo-inositol. Glutamine and myo-inositol were lower in both CC subgroups than in NC; glutamine and MQ, but not myo-inositol, differed between MCC and SCC.

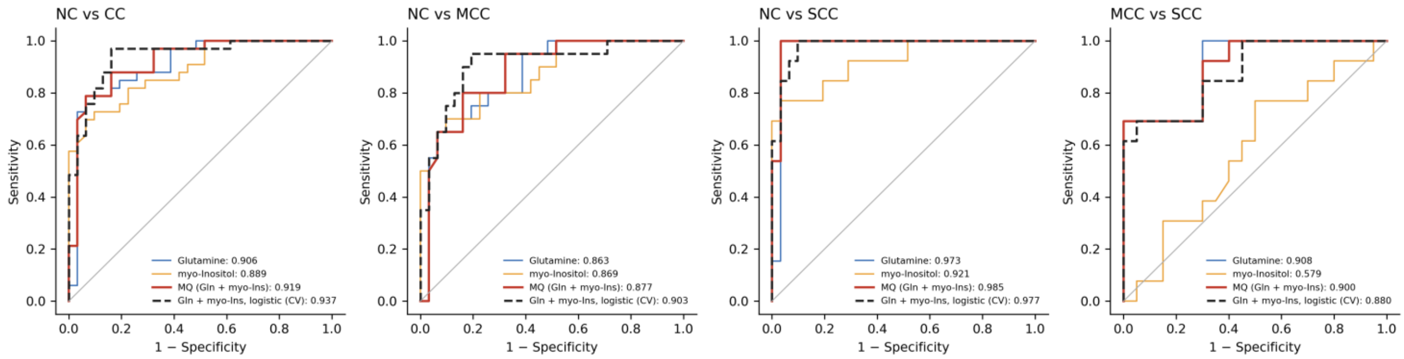

Figure S8. ROC curves for glutamine, myo-inositol and MQ. Panels show NC versus all CC, NC versus MCC, NC versus SCC, and MCC versus SCC. Solid lines: apparent (in-sample) ROC curves for single markers, with lower concentration indicating the cancer/larger-tumour class; dashed line: cross-validated predicted probabilities from a two-variable logistic model (glutamine + myo-inositol) averaged over 200 repeats of five-fold cross-validation. AUC values are given in the legends and Table S4.

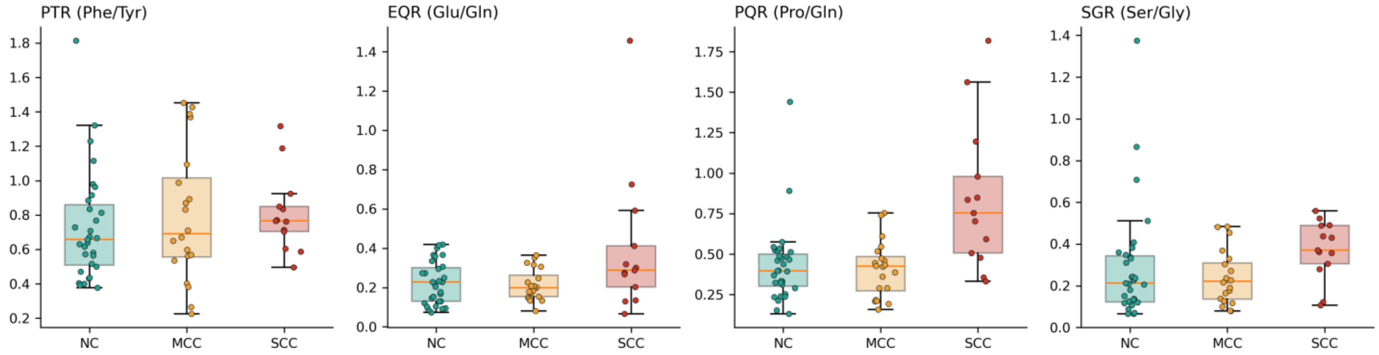

Figure S9. Metabolite ratio features. Phenylalanine/tyrosine (PTR), glutamate/glutamine (EQR), proline/glutamine (PQR) and serine/glycine (SGR) across NC, MCC and SCC. Boxes: median and interquartile range; points: individual participants. The ratios are exploratory and were not part of the 35-metabolite multiplicity family.

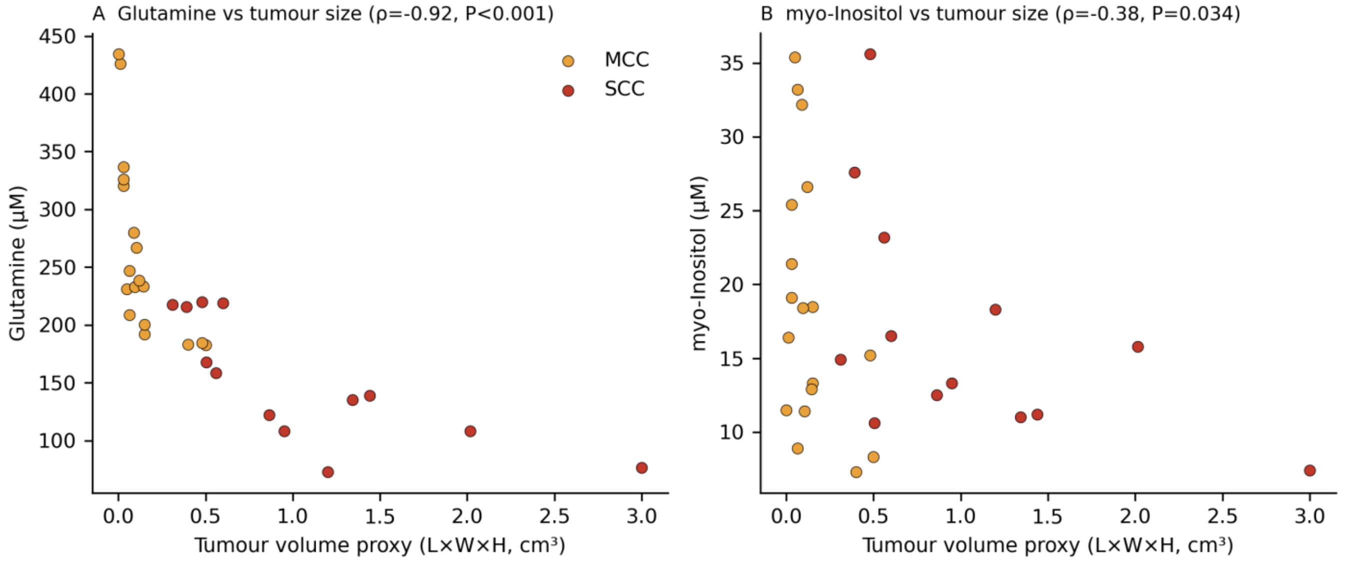

Figure S10. Relationship between tumour size and serum glutamine or myo-inositol within CC. (A) Glutamine and (B) myo-inositol versus a tumour-volume proxy (length × width × height, cm^3) for the 31 CC patients with complete tumour dimensions. Spearman correlation coefficients are given in the panel titles. Because the subgroups were defined from tumour length, this analysis is descriptive.

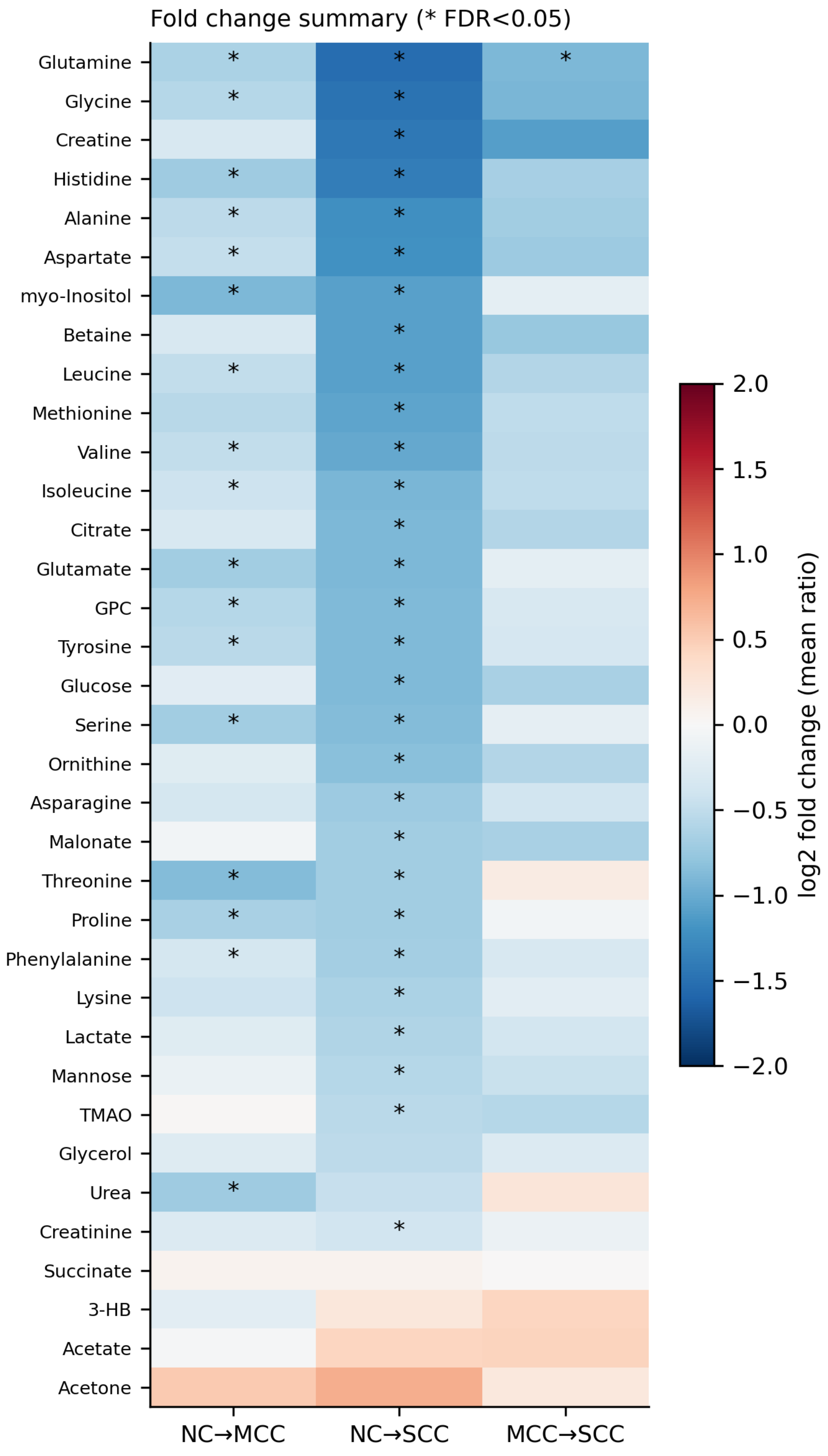

Figure S11. Fold-change summary across group comparisons. Heat map of log2 fold changes (ratio of means) for the 35 metabolites for NC→MCC, NC→SCC and MCC→SCC. Red indicates higher and blue lower concentration in the second group of each pair. Asterisks mark BH-FDR q < 0.05 (Mann–Whitney U test).

Supplementary Tables

Table S1. Complete results for all 35 primary serum metabolites (CC versus NC). Concentrations in µM (mean ± SD); one aspartate value was missing in NC (NC n = 30 for aspartate; not imputed for univariate tests). Hedges' g is CC minus NC (negative = lower in CC). Welch's t-test and Mann–Whitney U test q values are BH-FDR adjusted across the 35 metabolites. Age-adjusted fold change comes from a linear model of log2 concentration on group and age (n = 64), with BH-FDR across the 35 metabolites. 26 (Welch), 29 (Mann–Whitney) and 24 (age-adjusted) metabolites had q < 0.05. Rows are ordered by Welch P value. Means ± SD are given to two decimals (main-text Table 2 gives the same values to one decimal); Hedges' g confidence intervals use the main-text convention (g rounded to two decimals ± rounded 1.96 × SE).

Metabolite	CC (n=33) mean ± SD	NC (n=31) mean ± SD	Hedges' g [95% CI]	Welch BH q	Mann–Whitney BH q	Age-adjusted FC (CC/NC) [95% CI]	Age-adjusted BH q
Glutamine	229.72 ± 102.16	434.81 ± 123.80	-1.79 [-2.37, -1.21]	<0.001	<0.001	0.502 [0.405, 0.623]	<0.001
myo-Inositol	18.26 ± 8.81	35.77 ± 10.95	-1.75 [-2.33, -1.17]	<0.001	<0.001	0.469 [0.380, 0.579]	<0.001
Tyrosine	28.64 ± 10.73	45.58 ± 12.14	-1.46 [-2.01, -0.91]	<0.001	<0.001	0.604 [0.509, 0.717]	<0.001
Alanine	327.26 ± 151.81	555.43 ± 193.57	-1.30 [-1.84, -0.76]	<0.001	<0.001	0.565 [0.451, 0.707]	<0.001
Leucine	54.84 ± 23.77	88.65 ± 30.35	-1.23 [-1.77, -0.69]	<0.001	<0.001	0.597 [0.477, 0.747]	<0.001
Valine	127.48 ± 57.62	204.07 ± 70.31	-1.18 [-1.71, -0.65]	<0.001	<0.001	0.582 [0.464, 0.730]	<0.001
Isoleucine	46.88 ± 18.29	70.44 ± 22.68	-1.13 [-1.66, -0.60]	<0.001	<0.001	0.633 [0.516, 0.777]	<0.001
Glutamate	53.18 ± 21.25	90.82 ± 40.89	-1.15 [-1.68, -0.62]	<0.001	<0.001	0.589 [0.464, 0.748]	<0.001
Glycine	215.74 ± 129.25	391.98 ± 206.48	-1.02 [-1.54, -0.50]	<0.001	<0.001	0.556 [0.415, 0.746]	<0.001
Histidine	53.75 ± 25.30	103.03 ± 64.33	-1.01 [-1.53, -0.49]	0.001	<0.001	0.546 [0.436, 0.684]	<0.001
Aspartate	30.02 ± 19.91	49.91 ± 22.10	-0.94 [-1.46, -0.42]	0.001	<0.001	0.577 [0.440, 0.757]	<0.001
Serine	50.28 ± 20.31	85.00 ± 46.74	-0.96 [-1.48, -0.44]	0.001	<0.001	0.641 [0.495, 0.831]	0.003
Methionine	11.87 ± 6.47	19.69 ± 10.45	-0.90 [-1.41, -0.39]	0.002	0.003	0.755 [0.504, 1.129]	0.202
GPC	26.08 ± 13.08	41.87 ± 21.76	-0.88 [-1.39, -0.37]	0.003	0.003	0.662 [0.511, 0.857]	0.004
Glucose	3.92 ± 1.92	5.34 ± 1.45	-0.83 [-1.34, -0.32]	0.003	0.002	0.687 [0.554, 0.850]	0.002
Threonine	59.34 ± 26.13	102.99 ± 66.31	-0.87 [-1.38, -0.36]	0.003	<0.001	0.605 [0.451, 0.813]	0.003
Proline	112.44 ± 50.27	177.80 ± 97.46	-0.84 [-1.35, -0.33]	0.004	<0.001	0.628 [0.490, 0.806]	0.001
Lactate	1.68 ± 0.80	2.18 ± 0.53	-0.71 [-1.22, -0.20]	0.010	0.001	0.754 [0.630, 0.904]	0.005
Betaine	29.71 ± 20.60	44.41 ± 20.05	-0.71 [-1.22, -0.20]	0.010	<0.001	0.620 [0.481, 0.799]	0.001
Phenylalanine	22.88 ± 13.86	31.75 ± 13.05	-0.65 [-1.15, -0.15]	0.019	0.002	0.665 [0.519, 0.850]	0.003
Creatine	25.68 ± 19.26	40.68 ± 25.94	-0.65 [-1.15, -0.15]	0.019	0.011	0.599 [0.398, 0.900]	0.023
Lysine	35.77 ± 18.10	50.67 ± 26.74	-0.65 [-1.15, -0.15]	0.020	0.014	0.692 [0.540, 0.887]	0.008
Urea	61.30 ± 34.41	93.31 ± 61.47	-0.64 [-1.14, -0.14]	0.022	0.014	0.739 [0.536, 1.018]	0.083
Ornithine	30.87 ± 14.72	42.13 ± 21.48	-0.61 [-1.11, -0.11]	0.027	0.042	0.716 [0.549, 0.934]	0.023
Citrate	59.37 ± 44.31	85.18 ± 49.90	-0.54 [-1.04, -0.04]	0.046	0.044	0.700 [0.494, 0.991]	0.062
Creatinine	32.85 ± 17.62	40.83 ± 11.51	-0.53 [-1.03, -0.03]	0.048	0.008	0.787 [0.640, 0.968]	0.037
Mannose	49.25 ± 24.07	60.19 ± 18.20	-0.50 [-1.00, 0.00]	0.057	0.018	0.785 [0.633, 0.975]	0.042
Asparagine	17.21 ± 12.53	24.09 ± 15.69	-0.48 [-0.98, 0.02]	0.073	0.044	0.726 [0.527, 1.000]	0.067
Glycerol	97.43 ± 49.01	124.51 ± 69.15	-0.45 [-0.95, 0.05]	0.094	0.113	0.773 [0.579, 1.034]	0.102
Acetone	58.33 ± 67.91	38.41 ± 32.00	0.37 [-0.12, 0.86]	0.159	0.677	1.038 [0.622, 1.731]	0.912
Malonate	54.51 ± 33.80	65.88 ± 30.28	-0.35 [-0.84, 0.14]	0.182	0.049	0.851 [0.599, 1.208]	0.408
Acetate	65.50 ± 29.93	58.32 ± 26.95	0.25 [-0.24, 0.74]	0.346	0.460	1.079 [0.835, 1.395]	0.608
TMAO	5.18 ± 4.05	5.84 ± 3.34	-0.17 [-0.66, 0.32]	0.512	0.247	0.784 [0.526, 1.169]	0.266
Succinate	9.43 ± 5.03	8.99 ± 4.54	0.09 [-0.40, 0.58]	0.735	0.995	1.034 [0.803, 1.332]	0.841
3-HB	74.90 ± 65.58	75.85 ± 77.27	-0.01 [-0.50, 0.48]	0.958	0.811	0.980 [0.639, 1.505]	0.926

Table S2. Demographic and clinical characteristics by group (linked records). Categorical variables are n/available n (%); marital status was missing for one NC participant. P values: Kruskal–Wallis (age), permutation χ^2 (categorical, 5,000 permutations) and Mann–Whitney (tumour dimensions, MCC versus SCC). Age was also higher in all CC than NC (Mann–Whitney P = 0.029). HPV status in NC was recorded as negative for all 31 participants. Menopausal status, comorbidities, medication use, treatment status, FIGO stage, BMI and albumin were not available.

Characteristic	NC	MCC	SCC	P value	Test
N	31	20	13		
Age, years (mean ± SD)	47.0 ± 11.1	51.6 ± 12.1	54.3 ± 8.2	0.058	Kruskal–Wallis
Age, years (median, range)	46 (30–75)	52 (35–83)	55 (43–71)		
Age: NC vs CC (all cancer)				0.029	Mann–Whitney
Marital status: Yes, n (%)	28/30 (93%)	20/20 (100%)	13/13 (100%)	0.360	Permutation χ^2
Oral contraceptive: Yes, n (%)	10/31 (32%)	6/20 (30%)	6/13 (46%)	0.645	Permutation χ^2
Smoking status: Yes, n (%)	10/31 (32%)	5/20 (25%)	5/13 (38%)	0.689	Permutation χ^2
HPV: Positive, n (%)	0/31 (0%)	10/20 (50%)	6/13 (46%)	<0.001	Permutation χ^2
Tumour length, cm (mean ± SD)	—	0.7 ± 0.3	1.6 ± 0.3	<0.001	Mann–Whitney (MCC vs SCC)
Tumour width, cm (mean ± SD)	—	0.5 ± 0.2	1.2 ± 0.2	<0.001	Mann–Whitney (MCC vs SCC)
Tumour height, cm (mean ± SD)	—	0.3 ± 0.1	0.5 ± 0.2	0.001	Mann–Whitney (MCC vs SCC)

Table S3. Cross-validated PLS-DA summary. R^2Y: fitted variance explained; Q^2Y: repeated stratified five-fold cross-validation (five repeats); permutation P from 1,000 label permutations; AUC from the averaged cross-validated predicted scores (5 × 5-fold). This differs from the outer-fold mean AUC of the 10 × 5-fold PLS-DA evaluation reported in the main text (0.860 ± 0.090), which uses a different resampling scheme and summary.

Model	R2Y	Q2Y (5-fold CV x5)	Permutation P (1000)	CV AUC (predicted scores)
NC vs CC	0.61	0.34	0.001	0.85
NC vs MCC	0.56	0.24	0.003	0.82
NC vs SCC	0.80	0.65	0.001	0.98
MCC vs SCC	0.62	0.26	0.004	0.82

Table S4. ROC analysis, paired DeLong comparisons and cross-validated performance. Single-marker AUCs are apparent (in-sample) with stratified bootstrap 95% CI (2,000 replicates); cut-offs, sensitivities and specificities are exploratory in-sample estimates. Logistic models were evaluated by repeated five-fold cross-validation (200 repeats); the 95% range is the 2.5th–97.5th percentile across repeats; these two-variable and 35-metabolite models are distinct from the one-variable MQ logistic model (10 × 5-fold; mean AUC 0.912 ± 0.078) described in the main text. For NC versus all CC, the single-marker AUCs, bootstrap CIs, cut-offs, sensitivities and specificities are identical to main-text Table 3. Lower concentration indicates the cancer/larger-tumour class.

Comparison	Marker/model	Validation	AUC	95% CI	Cut-off (µM, apparent)	Sensitivity (apparent)	Specificity (apparent)
NC vs CC	Glutamine	single marker (apparent)	0.906	0.817–0.973	279.9	0.79	0.94
NC vs CC	myo-Inositol	single marker (apparent)	0.889	0.804–0.955	19.1	0.70	0.94
NC vs CC	MQ	single marker (apparent)	0.919	0.844–0.978	312.1	0.79	0.94
NC vs CC	Glutamine + myo-Inositol (logistic)	repeated 5-fold CV (200x)	0.937	0.923–0.947			
NC vs CC	All 35 metabolites (logistic, L2)	repeated 5-fold CV (200x)	0.833	0.789–0.870			
NC vs MCC	Glutamine	single marker (apparent)	0.863	0.757–0.953	279.9	0.65	0.94
NC vs MCC	myo-Inositol	single marker (apparent)	0.869	0.753–0.956	21.4	0.70	0.90
NC vs MCC	MQ	single marker (apparent)	0.877	0.765–0.959	358.2	0.80	0.84
NC vs MCC	Glutamine + myo-Inositol (logistic)	repeated 5-fold CV (200x)	0.903	0.879–0.921			
NC vs MCC	All 35 metabolites (logistic, L2)	repeated 5-fold CV (200x)	0.777	0.716–0.835			
NC vs SCC	Glutamine	single marker (apparent)	0.973	0.906–1.000	219.7	1.00	0.97
NC vs SCC	myo-Inositol	single marker (apparent)	0.921	0.813–1.000	18.3	0.77	0.97
NC vs SCC	MQ	single marker (apparent)	0.985	0.945–1.000	255.3	1.00	0.97
NC vs SCC	Glutamine + myo-Inositol (logistic)	repeated 5-fold CV (200x)	0.977	0.968–0.988			
NC vs SCC	All 35 metabolites (logistic, L2)	repeated 5-fold CV (200x)	0.972	0.955–0.983			
MCC vs SCC	Glutamine	single marker (apparent)	0.908	0.786–0.983	219.7	1.00	0.70
MCC vs SCC	myo-Inositol	single marker (apparent)	0.579	0.371–0.783	18.3	0.77	0.50
MCC vs SCC	MQ	single marker (apparent)	0.900	0.765–0.985	181.5	0.69	1.00
MCC vs SCC	Glutamine + myo-Inositol (logistic)	repeated 5-fold CV (200x)	0.880	0.831–0.915			
MCC vs SCC	All 35 metabolites (logistic, L2)	repeated 5-fold CV (200x)	0.760	0.677–0.838			

Paired DeLong comparison of correlated ROC curves (NC versus CC, same 64 participants)

Comparison	AUC 1	AUC 2	Difference	95% CI	P
MQ vs Glutamine	0.919	0.906	0.0132	-0.0031 to 0.0295	0.113
MQ vs myo-Inositol	0.919	0.889	0.0303	-0.0684 to 0.1290	0.547
Glutamine vs myo-Inositol	0.906	0.889	0.0171	-0.0928 to 0.1270	0.760

Table S5. Nested internal validation. In each training fold, the two metabolites with the smallest Mann–Whitney P values were selected and a logistic model was fitted (feature selection was therefore repeated inside every fold); 100 repeats of five-fold cross-validation. For MQ (which has no fitted weights), the ROC AUC is rank-based and unchanged by resampling, and the cut-off was set inside each training fold to obtain held-out sensitivity and specificity. The last three columns give the fraction of training folds in which glutamine, myo-inositol, or both were selected.

Comparison	AUC, nested top-2 model	Range	AUC, MQ	Range	Sensitivity (held-out)	Specificity (held-out)	Glutamine selected	myo-Inositol selected	Both selected
NC vs MCC + SCC	0.908	0.862–0.947	0.919	0.919–0.919	0.757	0.861	1.0	0.79	0.79
NC vs MCC	0.874	0.802–0.920	0.877	0.877–0.877	0.757	0.734	0.986	0.886	0.872
NC vs SCC	0.942	0.912–0.969	0.985	0.985–0.985	0.91	0.968	0.95	0.058	0.054
MCC vs SCC	0.841	0.794–0.879	0.9	0.900–0.900	0.644	0.856	1.0	0.0	0.0

Table S6. Tumour size and HPV status in relation to glutamine, myo-inositol and MQ within CC. Spearman correlation with tumour volume proxy (length × width × height) and length among the 31 CC patients with tumour dimensions; HPV comparison among 33 CC patients (HPV-positive n = 16; HPV-negative n = 17; Mann–Whitney U test). Exploratory; subgroups were defined from tumour length.

Variable	n	ρ vs volume	P	ρ vs length	P (length)
Glutamine	31	-0.92	<0.001	-0.94	<0.001
myo-Inositol	31	-0.38	0.034	-0.29	0.110
MQ	31	-0.92	<0.001	-0.93	<0.001

Variable	Median, HPV+ (μM)	Median, HPV− (μM)	P (Mann–Whitney)
Glutamine	225.3	200.4	0.221
myo-Inositol	17.8	15.2	0.428
MQ	260.8	213.7	0.145
